# An Improved Virtual Orbital Driven Similarity Renormalization Group Approach for Core-Ionized and Core-Excited States

**DOI:** 10.1021/acs.jctc.5c00457

**Published:** 2025-07-11

**Authors:** Meng Huang, Francesco A. Evangelista

**Affiliations:** † State Key Laboratory of Precision and Intelligent Chemistry, University of Science and Technology of China, Hefei, Anhui 230026, China; ‡ Department of Chemistry and Cherry Emerson Center for Scientific Computation, 1371 Emory University, Atlanta, Georgia 30322, United States

## Abstract

This work combines the multireference driven similarity renormalization group (DSRG) with a reference state obtained using improved virtual orbitals (IVOs) and generalized active space configuration interaction (GASCI) to model core-ionized and core-excited states without costly orbital optimizations. We test the accuracy of the resulting IVO-GASCI-DSRG method combined with three truncation levels across four data sets of molecules containing first-row elements (small molecules, potential energy surfaces, small-to-medium molecules, and X-ray absorption spectra). It is found that the IVO-GASCI-DSRG approach with an active space consisting of three GAS spaces and third-order perturbative corrections (IVO-GASCI[3]-DSRG-MRPT3) strikes the best balance between cost and accuracy. This method exhibits good agreement with the most accurate DSRG truncation scheme based on self-consistent orbitals on small-molecule benchmarks, and it is capable of accurately predicting the potential energy surfaces of core-excited and core-ionized states of CO, N_2_, and HF. To demonstrate the applicability of this method to medium-sized molecules, we simulate the X-ray absorption spectra of thymine and adenine using IVO-GASCI-DSRG-MRPT3, successfully reproducing key experimental spectral features.

## Graphical abstract

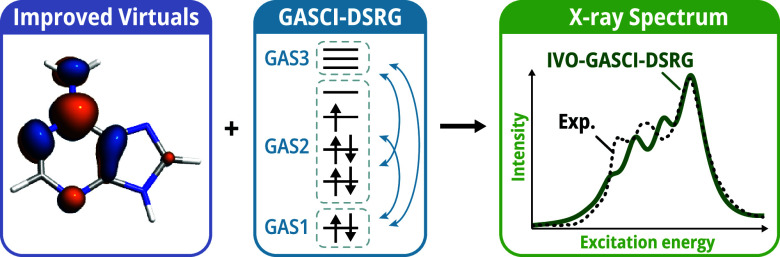

## 1 Introduction

The rapid advancement of X-ray spectroscopy techniques^1–9^ has increased the need for reliable and efficient theoretical models that can accurately predict core-ionized and core-excited states. However, developing electronic structure methods for these states presents significant challenges that arise from two factors.^10,11^ First, core-excited and core-ionized states are found at significantly higher energies than valence states, complicating the application of conventional algorithms that diagonalize Hamiltonian matrices to extract only a limited number of low-energy roots. Second, accurate modeling of these states necessitates accounting for orbital relaxation and polarization effects induced by the presence of a core hole.

Among the various theoretical approaches developed for core-ionized and core-excited states,^12–109^ methods based on Multi-Configurational Self-Consistent Field (MCSCF) offer a straightforward means to address the challenges posed by the high energy of the states and the need for orbital relaxation treatment.^101–109^ In particular, the Restricted and Generalized Active Space Self-Consistent Field (RASSCF/GASSCF) methods^110–112^ and their extension including second-order perturbative corrections (e.g., RASPT2)^113,114^ approximate high-energy core-excited states by limiting the number of electrons allowed in core orbitals. Multiconfigurational Pair-Density Functional Theory (MC-PDFT),^115^ which combines the accuracy of multiconfigurational methods for treating strong electron correlation with the computational efficiency of density functional theory through the use of electron pair density, has been also applied on evaluating core-excited states of hydrated transition metal ions^107,108^ and organic molecules.^109^ These multireference strategies enable targeting core-ionized and core-excited states by computing several of the lowest eigenvalues among the RAS/GAS determinants. Furthermore, the RASSCF/GASSCF approach optimizes the molecular orbitals in the presence of a core hole, thereby incorporating the static correlation associated with core relaxation effects. While other single-reference methods can address core relaxation effects or target high-energy eigenvalues through ΔSCF^116^ or Core–Valence Separation (CVS) techniques,^117^ MCSCF-based methods can additionally target general states with near-degenerate valence orbitals. This capability is essential for accurately modeling open-shell systems, including molecules containing transition metals or undergoing bond-breaking processes.

Despite their desirable features, MCSCF-based methods are more challenging to use than single-reference ones. First, the cost to optimize the orbitals is higher, owing to a combination of slower convergence and the additional cost of the configuration interaction step. Second, although RASSCF methods are theoretically capable of converging to the Full Configuration Interaction (FCI) limit as the active space expands, the inclusion of additional virtual orbitals can lead to the appearance of local minima in the orbital optimization landscape, thereby potentially yielding unreliable results.^118^ Furthermore, in potential energy scans, MCSCF methods may struggle in regions with near-degenerate electronic states or encounter discontinuities due to the swapping of orbitals between active and virtual/core spaces. Moreover, an accurate description of core-excited states requires orbitals distinct from those used for ground states, complicating the evaluation of transition properties. These challenges highlight the need to develop alternative methods to MCSCF for evaluating core-ionized and core-excited states.

Improved Virtual Orbital (IVO) methods^119–121^ have been developed to partially incorporate core-relaxation effects, and are widely utilized in the study of core-excited and core-ionized states.^12,122–125^ Specifically, IVO-based methods generate a set of virtual orbitals by diagonalizing the Fock operator corresponding to a core-ionized state, thereby providing an approximate inclusion of core-relaxation effects. This step is followed by a CI calculation to generate a multireference description of specific core-excited or core-ionized states. The IVO-RASCI approach has demonstrated some practical benefits in the description of core-excited states^126,127^ compared to the RASSCF approach.^101–105^ First, it eliminates the need for multideterminantal orbital optimization, which is more prone to convergence issues than mean-field methods, and is generally more expensive due to the need to perform multiple CI computations. This advantage is particularly relevant when targeting a large number of excited states. Second, by avoiding orbital optimization, IVO-RASCI eliminates the potential for a RASSCF computation to converge to a local minimum or an unphysical solution, or worse, a solution that disappears along a reaction path.^128^ These issues generally hinder the consistent application of multireference methods to problems that involve sampling multiple geometries or reaction paths. Lastly, because the IVO basis is not optimized self-consistently, expanding the active space leads to more systematic convergence toward the full configuration interaction limit. Nevertheless, the use of IVO does not eliminate the active space selection problem and the potential for orbitals to rotate out of the active space while scanning a series of geometries. Another potential issue of IVOs indicated by prior studies^12,122–125^ is the partial accounting of core-relaxation effects, which can result in significant inaccuracies (up to 10 eV) in the computed transition energies for core-excited states.

In this work, we address the accuracy issues of the IVO-GASCI approach for core-excited and core-ionized state energies using our driven similarity renormalization group (DSRG) approach.^129,130^ Previous work focusing on valence excited states^131–133^ has shown that combining IVO-CASCI with a subsequent treatment of dynamical correlation holds great potential. Our methodology leverages the efficient IVO-GASCI approach (which includes as a special case IVO-RASCI) to incorporate core relaxation effects, while the MR-DSRG method accounts for the remaining dynamical correlation. We evaluate the accuracy of the IVO-GASCI approach combined with various DSRG truncation schemes for computing core-ionization and core-excitation energies of small molecules, potential energy curves of core-ionized and core-excited states of diatomic molecules, and the X-ray absorption spectra (XAS) of thymine and adenine. This methodology achieves an excellent balance between efficiency and accuracy when evaluating core-excited and core-ionized states. This paper is structured as follows: Section 2 provides an overview of the theoretical framework, including the IVO approach, the GASCI approach, and the MR-DSRG theory. In Section 3, we provide details of the computations performed in this study. In Section 4, we present and discuss our results, evaluating its accuracy compared to the results from DSRG calculations based on a GAS self-consistent-field reference state and experimental results. We specifically investigate how the accuracy of IVO-GASCI-DSRG is affected by the choice of active space. Finally, Section 5 concludes the paper with a summary of our findings and potential future directions for this research.

## 2 Theory

In this section, we present an overview of the IVO approach and MR-DSRG theory for calculating core-ionized and core-excited states. The IVO approach generates orbitals that efficiently incorporate core relaxation effects, while the truncated MR-DSRG method further refines these orbitals and accounts for missing dynamical electron correlation.

### 2.1 Improved Virtual Orbitals

We implemented the IVO approach following the formalism described in ref 134. The IVO procedure begins with either a Restricted Hartree–Fock computation or its Open-shell variant (ROHF) when dealing with open-shell molecules. Focusing on closed-shell ground states, the Fock matrix is defined as

Fpq=⟨ϕp|h^+∑kocc(2J^k−K^k)|ϕq⟩ 1

where ϕ_*p*_ and ϕ_*q*_ denote Hartree–Fock (spatial) orbitals, and *ĥ*, *Ĵ*_*k*_, and *K̂*_*k*_ are the one-electron operator and the Coulomb and exchange contributions from orbital ϕ_*k*_, respectively. Assuming the use of canonical orbitals, the Fock matrix is diagonal:

$$F_{pq}=\delta_{pq}\epsilon_{p}$$ 2

with the diagonal elements ϵ_*p*_ being the RHF orbital energies. Upon removal of an electron from a doubly occupied orbital ϕ_*i*_, the IVO approach constructs a new set of virtual orbitals, ϕ_*a*_′, as a linear combination of the original virtual orbitals ϕ_*a*_. This is accomplished by diagonalizing the virtual block of the effective Fock matrix (*F̂*′), with matrix elements in the RHF orbital basis given by

$$F_{ab}^{\prime }=F_{ab}+\langle \phi_{a}|-{\hat{J}}_{i}+2{\hat{K}}_{i}|\phi_{b}\rangle$$ 3

The eigenvectors of *F*′, arranged in the matrix *C*′, transform the canonical RHF virtual orbitals into IVOs:

$$\phi_{a}^{\prime }=\overset{\mathrm{vir}}{\underset{b}{\sum }}\phi_{b}C_{ba}^{\prime }$$ 4

with corresponding eigenvalues ϵ_*a*_′ being the IVO orbital energies. The original RHF occupied orbitals remain unchanged and orthogonal to the new improved virtual orbitals. For molecules with symmetric core orbitals, such as homonuclear diatomic molecules or benzene, IVOs are generated by averaging all symmetric core orbitals using

$$F_{ab}^{\prime }=F_{ab}+\frac{1}{N_{c}}\sum_{i}^{N_{c}}\langle \phi_{a}|-{\hat{J}}_{i}+2{\hat{K}}_{i}|\phi_{b}\rangle$$ 5

where *N*_c_ is the number of identical core orbitals. The ROHF case is handled in a similar way, starting from the α Fock matrix (as defined in Unrestricted Hartree–Fock), and proceeding to account for the removal of one α electron from a core orbital.

### 2.2 Generalized Active Space Configuration Interaction

After the IVO step, we generate GASCI multideterminantal wave functions that model the ground state (Ψ_0_) and core-excited or core-ionized states (Ψ_α_, α = 1, 2,..., *m*). These states are of the form:

$$\left|\Psi_{\alpha }\right\rangle =\overset{\mathrm{GAS}}{\underset{\mu }{\sum }}C_{\alpha }^{\mu }\left|\Phi_{\mu }\right\rangle$$ 6

where |Φ_μ_⟩ is a determinant that satisfies specified occupation restrictions and *C*_α_^μ^ its corresponding coefficient. In the GASCI approach the active space is partitioned into several subspaces (denoted as GAS*n*, where *n* = 1, 2,...). We always assign the core orbital ϕ_*i*_ to the GAS1 subspace and valence orbitals to the GAS2 subspace. An optional GAS3 subspace can be introduced to consider additional valence orbitals. With this partitioning, we target specific core-excited and core-ionized states by restricting the electron count in the various GAS subspaces. For example, to target the carbon K-edge core excitations in CO using a full valence active space, we include the C 1s-like orbital in the GAS1 subspace, restricting its occupation to 1, and distribute the remaining 13 electrons in the GAS2 subspace (which would span the 2s and 2p orbitals of C and the 1s, 2s, and 2p orbitals of O). The occupation of the GAS2 subspace would be restricted to 12 electrons to obtain the analogous core-ionized states. The same set of IVOs is used for both core-excited and core-ionized states when electrons are excited or ionized from identical core orbitals. To target core-ionized and core-excited states originating from different core orbitals, the IVO and GASCI procedures are performed separately for states that promote an electron from different core orbitals, thereby enabling a parallel computation of the spectrum.

### 2.3 Multireference Driven Similarity Renormalization Group

By incorporating dynamical electron correlation through multireference DSRG theory,^135^ we can further improve the description of core-excited and core-ionized states based on zeroth-order GASCI reference states and IVO orbitals. In MR-DSRG, an effective Hamiltonian is constructed via a unitary transformation:

$$\hat{H}\to \overset{®}{H}=e^{-\hat{A}}\hat{H}e^{\hat{A}}$$ 7

where the anti-Hermitian operator *Â* = *T̂* – *T̂* ^†^ is a generalized form of the coupled cluster excitation operator *T̂*. This transformation decouples *H̅* by eliminating the off-diagonal couplings (denoted with *H̅* ^*N*^) between determinants inside and outside the GAS space. Once this decoupling is achieved (i.e., *H̅* ^*N*^ = 0), the exact eigenvalues for a set of states can be obtained by diagonalizing *H̅* within the GAS determinant space.

To avoid the intruder state problems when solving *H̅* ^*N*^ = 0 (i.e., numerical issues caused by small denominators), the DSRG amplitudes are determined by solving a series of many-body equations:

H®N(s)=R^(s) 8

In this equation, the flow parameter *s* controls the suppression of excitations with small energy denominators responsible for the intruder state problem. The source operator *R̂*(*s*) appearing on the right-hand side of eq 8 satisfies *R̂* (*s*) → 0 as *s* → *∞*. After solving the DSRG equation, the state-specific DSRG energy is obtained by diagonalizing the DSRG Hamiltonian [*H̅*(*s*)] within the GAS determinant space:

$$\overset{®}{H}(s)|\Psi^{\prime }\rangle =E^{\prime }(s)|\Psi^{\prime }\rangle$$ 9

here, *E′*(*s*) and |Ψ′⟩ represent the relaxed energy and reference state, respectively.^129,130,136–139^ In the state-averaged DSRG framework, multiple solutions are obtained by solving eq 9. Note that the state-averaged variant of MR-DSRG is capable of handling multiple states at once, including near-degenerate states and conical intersections.^135^ Studies have been performed to identify an optimal value of the flow parameter in applications to valence-^140^ and core-excited states.^105,141^

This study uses two MR-DSRG approaches to evaluate core-ionized and core-excited states. The first approach employs perturbative approximations of MR-DSRG, truncated at second and third-order^136,138^ (denoted as DSRG-MRPT*n*, with *n* = 2, 3). In these methods, the zeroth-order Hamiltonian is the diagonal part of the GASCI generalized Fock matrix expressed in a semicanonical basis. Efficient implementations of both DSRG perturbative methods^139^ require up to three-body reduced density matrices of the GASCI reference states. The second approach is the linearized MR-DSRG with one- and two-body operators [MR-LDSRG(2)]. While this scheme goes beyond perturbation theory and is analogous to the unitary coupled-cluster method with singles and doubles (UCCSD), its accuracy is limited to second order. For the calculations performed in this study, where the size of the active space (*N*_A_) is much smaller than the number of virtual orbitals (*N*_V_), the DSRG-MRPT2 method scales as *O*(*N*_C_^2^ *N*_V_^2^) where *N*_C_ is the number of core orbitals. The DSRG-MRPT3 scales as *O*(*N*_C_^2^ *N*_V_^4^), while the LDSRG(2) scales as *O*(*N*_C_^2^ *N*_V_^2^ *N* ^2^) (iterative) where *N* = *N*_A_ + *N*_C_ + *N*_V_ is the total number of orbitals. For large active spaces, the leading term for all three methods scales as *O*(*N*_A_^6^ *N*_V_). When combined with an IVO-GASCI reference, the computational cost of these methods is identical to that of our earlier work on the GASSCF-DSRG approach, with the only reduction in cost coming from the need to perform only a GASCI computation instead of one or multiple GASSCF computations.

## 3 Computational Details

For GASCI-DSRG calculations, we obtain IVOs from a Restricted Hartree–Fock (RHF) calculation. Subsequently, we perform GASCI-DSRG calculations using the IVO orbitals to obtain energies for ground, core-excited, and core-ionized states by varying the GAS restrictions, charges, and spin multiplicities. In our calculations, we utilized two different types of active spaces. One type, denoted as GAS[2]­(*n*_1_, *n*_2_), partitions the active space into two GAS subspaces containing *n*_1_ and *n*_2_ orbitals, respectively. For core-excited or core-ionized states, GAS1 contains all the core orbitals of a specific element, with the number of electrons constrained to be 2*n*_1_ – 1. For the corresponding ground state, we force the number of electrons to be 2*n*_1_. This is the general approach we have used for GASSCF-DSRG calculations.^105,141–143^ The other scheme, denoted as GAS[3]­(*n*_1_, *n*_2_, *n*_3_), partitions the active space into three GAS subspaces containing *n*_1_, *n*_2_, and *n*_3_ orbitals. In this case, the number of electrons in GAS1 is also fixed to be 2*n*_1_ – 1, while the number of electrons in GAS3 can vary between 0 and 2, as in the RAS approach. Accordingly, we refer to these two types of GASCI-DSRG calculations as GASCI[2] and GASCI[3]. Since all previous GASSCF-MR-DSRG calculations^105,141,143^ were performed using two active spaces, we will refer to these simply as GASSCF-MR-DSRG. All calculations were performed using the PSI4 ^144^ and Forte^145^ software packages.

We evaluated 42 core-ionization and core-excitation energies of 14 small molecules, each containing one or two first-row elements,^67^ to test the accuracy of the IVO-GASCI-DSRG approaches. The vertical transition energies were computed using the DSRG-MRPT2, DSRG-MRPT3, and MR-LDSRG(2) levels of theory with the cc-pCVQZ-DK basis set. Relativistic effects were included via the spin-free exact-two-component (X2C)^146^ scalar one-electron treatment. The state-specific DSRG formalism was employed for all cases except for core-excitation energies corresponding to 1s → π* transitions in linear molecules. For these cases, we averaged the degenerate excitations over the largest Abelian subgroup while preserving the linear symmetry of the orbitals.

We used molecular geometries from Liu et al.,^67^ where diatomic geometries were taken from experimental data, and polyatomic geometries were optimized at the SFX2C-1e-CCSD­(T)/cc-pCVQZ level of theory.^147,148^ For the flow parameter *s*, in our calculations we selected *s* = 1 *E*_h_^–2^ for the DSRG-MRPT2/3 and *s* = 0.5 *E*_h_^–2^ for the MR-LDSRG(2), consistent with our previous studies.^105,140,141^ These parameters were also used to evaluate the potential energy surfaces of the ground, core-excited, and core-ionized states of CO, N_2_, and HF using only the DSRG-MRPT3 level of theory. This choice is based on the observation that DSRG-MRPT3 yields results closely matching those from the much more expensive MR-LDSRG(2) method in potential energy surface scans of diatomic molecules.^105^ These scans were performed from 0.8 to 2.0 Å with a step size of 0.05 Å.

To assess the performance of our IVO-GASCI-DSRG methodologies on moderately large organic molecules, we employed the CORE65 ^32^ and XABOOM^149^ benchmark data sets. Core-ionization and core-excited energies for first-row elements were computed at the IVO-GASCI-DSRG-MRPT3 level using the cc-pVQZ basis set, with relativistic effects excluded from the calculations. The molecular structures from CORE65 were taken as previously optimized using density functional theory with tier 2 numeric atom-centered orbitals.^150^ The molecular geometries from the XABOOM set are optimized at the frozen-core MP2/cc-pVTZ level of theory.^149^ For molecules exhibiting multiple excitations or ionizations, we utilized the state-averaged variant of the DSRG approach.^135,141^ CORE65 and XABOOM computations utilized a flow parameter of *s* = 1 *E*_h_^–2^.

We simulated the X-ray absorption spectra of thymine and adenine using the IVO-GASCI-DSRG approach, specifically at the DSRG-MRPT3 level of theory with the cc-pVQZ basis set. The molecular geometries were obtained from the XABOOM set,^149^ optimized at the frozen-core MP2/cc-pVTZ level of theory. The O, N, and C K-edge of thymine XAS were calculated with a GAS[3]­(1,5,22) active space while the N and C K-edge of adenine XAS were calculated with a GAS[3]­(1,4,17) active space. Separate IVO calculations were performed for each 1s core orbital of non-hydrogen atoms in both molecules, following the separate-core approach used in our previous GASSCF-DSRG calculations on this set.^141^ The oscillator strength of each transition is directly computed from the transition dipole moments evaluated from the GASCI states, as we employed the same set of orbitals for both the ground and core-excited states for each IVO calculation.

## 4 Results and Discussion

### 4.1 Core-Ionization and Core-Excitation Energies of Small Molecules

We begin by evaluating the effect of using IVO orbitals in GASCI-DSRG theory by computing the core-ionization and core-excitation energies of small molecules using a full active space comprising all core and valence orbitals. Error statistics were computed with respect to GASSCF-MR-LDSRG(2) results—the most accurate truncation level for multireference DSRG theory^137^—using the same active space to provide a direct comparison between the IVO and GASSCF approaches. The Mean Absolute Error (MAE) and Standard Deviation (STD) for the IVO-GASCI[2] theory are presented in Table 1, alongside GASCI-DSRG results based on canonical (Hartree–Fock) orbitals (denoted as GASCI[2]-DSRG), GASSCF-DSRG-MRPT2, and GASSCF-DSRG-MRPT3 results. For this data set, the GASSCF-MR-LDSRG(2) method exhibits a mean absolute error (MAE) of 0.55 eV relative to experimental data^151–160^ across all core-excitation and core-ionization energies. This relatively larger error for small molecules has been previously investigated,^105,143^ with findings suggesting that error cancellation effects may result from vibrational contributions and limited experimental resolution. For completeness, errors relative to experimental transition energies are listed in Table S8 of the Supporting Information. These exhibit trends similar to those in Table 1.

**Table 1:** Error Statistics (in eV, MAE = Mean Absolute Error, STD = Standard Deviation) for Core-Ionization and Core-Excitation Energies of Small Molecules Computed by GAS-DSRG Methods Using R­(O)­HF, IVO, and GASSCF Orbitals^a^

	overall			core ionization			core excitation		
	MAE	RMSE	STD	MAE	RMSE	STD	MAE	RMSE	STD
R(O)HF-GASCI[2]-DSRG-MRPT2	4.73	5.88	3.54	4.73	5.90	3.61	4.72	5.86	3.55
R(O)HF-GASCI[2]-DSRG-MRPT3	1.78	2.46	1.75	1.33	2.07	1.67	2.22	2.80	1.74
R(O)HF-GASCI[2]-MR-LDSRG(2)	1.07	1.24	0.69	0.89	1.04	0.57	1.24	1.40	0.77
R(O)HF-GASCI[3]-DSRG-MRPT2	3.91	4.74	2.72	3.97	4.83	2.81	3.84	4.65	2.69
R(O)HF-GASCI[3]-DSRG-MRPT3	0.89	1.32	1.24	0.86	1.20	1.21	0.93	1.43	1.21
R(O)HF-GASCI[3]-MR-LDSRG(2)	0.72	0.83	0.44	0.68	0.76	0.37	0.76	0.88	0.50
IVO-GASCI[2]-DSRG-MRPT2	1.48	2.29	1.78	1.81	2.66	2.00	1.16	1.84	1.50
IVO-GASCI[2]-DSRG-MRPT3	0.62	0.97	0.79	0.55	0.92	0.81	0.69	1.02	0.77
IVO-GASCI[2]-MR-LDSRG(2)	0.48	0.57	0.32	0.39	0.47	0.27	0.57	0.65	0.35
IVO-GASCI[3]-DSRG-MRPT2	0.81	1.13	0.84	0.84	1.17	0.90	0.78	1.09	0.80
IVO-GASCI[3]-DSRG-MRPT3	0.27	0.45	0.42	0.27	0.47	0.46	0.27	0.33	0.38
IVO-GASCI[3]-MR-LDSRG(2)	0.33	0.42	0.27	0.38	0.44	0.22	0.28	0.40	0.31
GASSCF-DSRG-MRPT2	0.29	0.47	0.38	0.15	0.23	0.19	0.43	0.63	0.47
GASSCF-DSRG-MRPT3	0.10	0.18	0.18	0.08	0.11	0.11	0.12	0.23	0.22

a Errors are relative to GASSCF-MR-LDSRG(2) results. Results are reported for both a small active space with 2 GAS spaces (GASCI[2]) and a larger one with 3 GAS spaces (GASCI[3]) when using R­(O)­HF and IVO orbitals. For GASSCF, only the 2 GAS spaces results are reported. All results were computed using the cc-pCVQZ-DK basis and X2C one-electron scalar relativistic corrections.

A detailed analysis in the Supporting Information on the absolute DSRG energy demonstrates that the IVO approach primarily impacts the core-ionized and core-excited states. In contrast, the ground state energies remain relatively consistent with different orbital choices. The errors in core-ionization and core-excitation energies of small molecules notably decrease with more accurate treatments of static and dynamical correlations. When using canonical RHF orbitals, the GASCI[2] reference yields a substantial MAE of 4.73 eV at the DSRG-MRPT2 level, which decreases to 1.78 eV at the DSRG-MRPT3 level. Even with the highly accurate dynamical treatment provided by MR-LDSRG(2), calculations based on canonical orbitals and a GASCI[2] reference still show an MAE of 1.07 eV. The IVO treatment, which partially accounts for core-relaxation effects, reduces these errors to 1.48 eV for DSRG-MRPT2, 0.62 eV for DSRG-MRPT3, and 0.48 eV for the highly accurate MR-LDSRG(2) theory. We also observe that, under DSRG-MRPT3 and MR-LDSRG(2) theories, IVO orbitals achieve better agreement in core-ionization energies (0.55 and 0.39 eV MAE, respectively) than in core-excitation energies (0.69 and 0.57 eV MAE, respectively). It is important to point out that since the IVO-GASCI-based approach is an approximation to the GASSCF-based parent method, it cannot generally yield results that are systematically more accurate than the latter (apart from spurious error cancellation). Indeed, when using a GASSCF reference, which incorporates a full treatment of orbital relaxation, the MRPT2 and DSRG-MRPT3 levels of theory yield results closer to the LDSRG(2) for small molecules, with minimal deviations from the reference LDSRG(2) values of 0.29 and 0.10 eV, respectively.

The IVO approximation provides a reasonable sub-eV error in approximating the GASSCF-DSRG results, comparable to those obtained from multireference many-body expansion methods such as MR-ADC(2)-X.^161^ Compared to experimental values, core-ionization and core-excitation energies computed using various IVO-GASCI[2]-DSRG methods range from 1.01 to 1.26 eV. These errors are generally smaller than those obtained from EOM­(IP)-CCSD results;^57,65,67,149^ however, they do not achieve the accuracy of CC methods that incorporate additional electron correlation (e.g., EOM-CCSDT^57,65,67^ ) or explicitly account for core-relaxation effects.^68^

To further analyze the excitation energy errors, we consider the error distributions of the IVO-GASCI[2]-DSRG core-ionization and core-excitation energies, which are plotted in Figure 1. The corresponding error distributions relative to experimental values are shown in Figure S1 of the Supporting Information. An analysis of the error distributions suggests that most errors originate from a few outliers, such as F_2_, CO_2_, H_2_O, and HF. In the cases of F_2_ and CO_2_, it is noteworthy that these errors are already significant when using GASSCF orbitals, indicating that the dynamical correlation treatment is crucial for accurately describing the core-ionization and core-excitation processes. On the other hand, for small molecules like H_2_O and HF, the valence active space comprises only two and one virtual orbitals, respectively. Therefore, in these cases, we attribute the significant deviations to the inclusion of a limited number of virtual orbitals in the active space.

**Figure 1:**
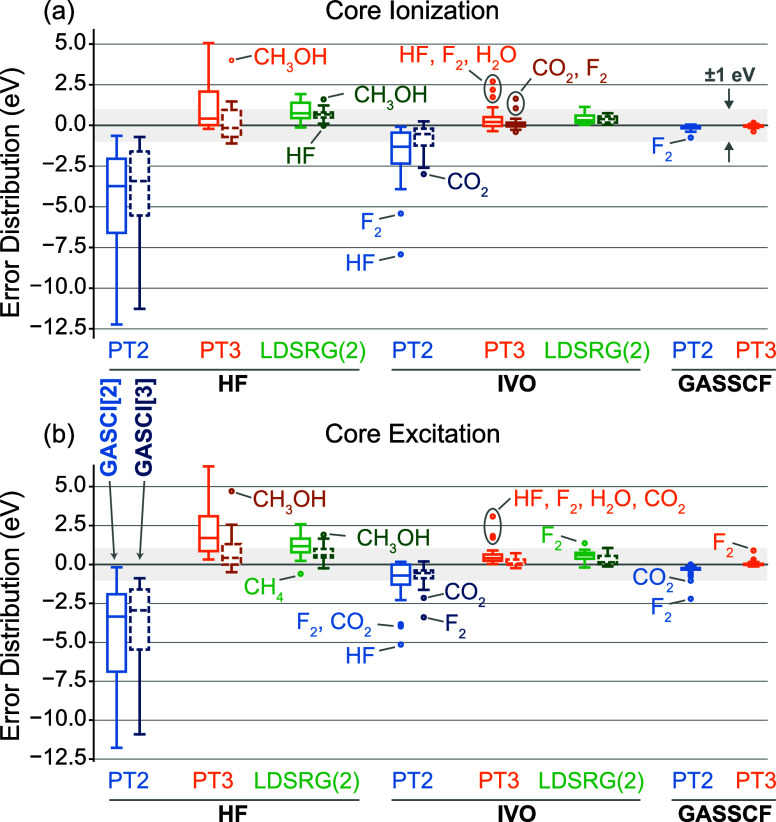
Boxplots for the error distribution of (a) core-ionization and (b) core-excitation energies of small molecules computed with GAS-DSRG theories using canonical R­(O)­HF, IVO, and GASSCF orbitals, relative to the GASSCF-MR-LDSRG(2) results. Results are reported for both a small active space with 2 GAS spaces (GASCI[2], left light box) and a larger one with 3 GAS spaces (GASCI[3], right dark box) for R­(O)­HF and IVO orbitals. For GASSCF, only the results for the GASCI[2] are reported. All results were computed using the cc-pCVQZ-DK basis and X2C one-electron scalar relativistic corrections. For GASSCF results, the orbitals are separately optimized for ground and core-ionized/core-excited states.

To fully account for the static correlation introduced by the IVO approach, we will next explore the impact of including additional optimized virtual orbitals in the active space. However, increasing the size of the active space significantly raises the computational cost of the GASCI step. To mitigate this cost and retain the accuracy of a full active space comprising all core and valence orbitals, we extend the active space to include the next three to four virtual orbitals (ranked by orbital energy) and partition the active space into three subspaces. Specifically, the GAS1 subspace contains only core orbitals (and is restricted to having one hole), while the GAS2 subspace includes all occupied orbitals plus a small set of empty orbitals required to describe the target core-excited state (for core-excitation calculations). No restrictions are applied to the GAS2 subspace. The GAS3 subspace consists of the remaining empty active orbitals, and its occupation can range from zero to two. The MAE and STD of the GASCI-DSRG results utilizing a larger active space and the three-space GAS approach (denoted with GASCI[3]) with respect to the GASSCF-MR-LDSRG(2) results are shown in Table 1. The corresponding error statistics and contributions relative to the experimental results are shown in Table S8 and Figure S1 of the Supporting Information. At all levels of theory, including those based on canonical orbitals, the GASCI[3] approach exhibits significantly lower MAE and STD than GASCI[2] for the calculated core-excited/core-ionized states, illustrating the benefit of increasing the size of the active space, even if the determinant space is truncated. Once again, going from canonical orbitals to IVOs increases the accuracy of the GASCI[3]-DSRG approach, reducing the MAE from 1.48/0.62/0.48 eV to 0.81/0.27/0.33 eV at the DSRG-MRPT2, DSRG-MRPT3, and LDSRG(2) levels of theory, respectively.

A detailed examination of the error distribution (Figure 1) indicates that IVO-GASCI[3]-DSRG-MRPT3 still faces challenges in accurately computing core-excited and core-ionized states for molecules such as CO_2_ and F_2_, where significant discrepancies exist among different DSRG levels even when using GASSCF orbitals. Expanding the active space can further reduce these errors. For F_2_, increasing the total active space size from 14 to 16 decreases the core-ionization and core-excitation energy errors from 1.06/0.70 eV to 0.68/0.22 eV, respectively. Similarly, for CO_2_ (O K-edge), increasing the total active space size from 18 to 21 reduces the core-ionization and core-excitation energy errors from 1.64/1.42 to 1.11/0.73 eV, respectively. These findings indicate that IVO-GASCI[3]-DSRG-MRPT3 with a slightly expanded active space (partitioned into three subspaces) achieves an MAE of less than 0.3 eV in simulating core-excited and core-ionized states, making it an outstanding alternative to GASSCF-based methods or the more computationally demanding MR-LDSRG(2) approaches.

### 4.2 Potential Energy Surface Calculations

We have shown that the IVO approach, especially in combination with the DSRG-MRPT3 level of theory, provides a compelling alternative to GASSCF-DSRG approaches for computing core-ionization and core-excitation energies for molecules at their equilibrium geometries. However, the primary use case of the multireference approaches developed here is in potential energy surface scans, where they are likely to outperform most single-reference approaches. Therefore, our next tests examine the impact of IVOs on the accuracy of potential energy curves of ground, core-ionized, and core-excited states of three representative diatomic molecules (CO, N_2_, and HF). These computations cover both the cases of ground states dominated by a single determinant (near the equilibrium bond length) and highly multideterminantal wave functions (at stretched geometries).

We consider the O K-edge core-excited (O 1s → π*) and core-ionized states of CO to demonstrate the significance of our approach in simulating molecules at stretched geometries. Figure 2a,b display the potential energy curve errors (relative to GASSCF-DSRG-MRPT3) for core-excited and core-ionized states for DSRG-MRPT3 theories based on different orbitals and active space partitions. Figure S2 in the Supporting Information shows the corresponding potential energy curves, confirming that the IVO-GASCI approach generates continuous, smooth potential energy surfaces. Previous research has shown minimal differences between GASSCF-DSRG-MRPT3 and GASSCF-MR-LDSRG(2) for these diatomic molecules.^105^ Results based on canonical Hartree–Fock orbitals (shown with empty circles) deviate significantly from the GASSCF curve, with errors that strongly depend on the bond distance. Including three additional orbitals in the active space did not significantly enhance accuracy; moreover, using more than two GAS spaces resulted in an abnormal shoulder in the potential energy curve.

**Figure 2:**
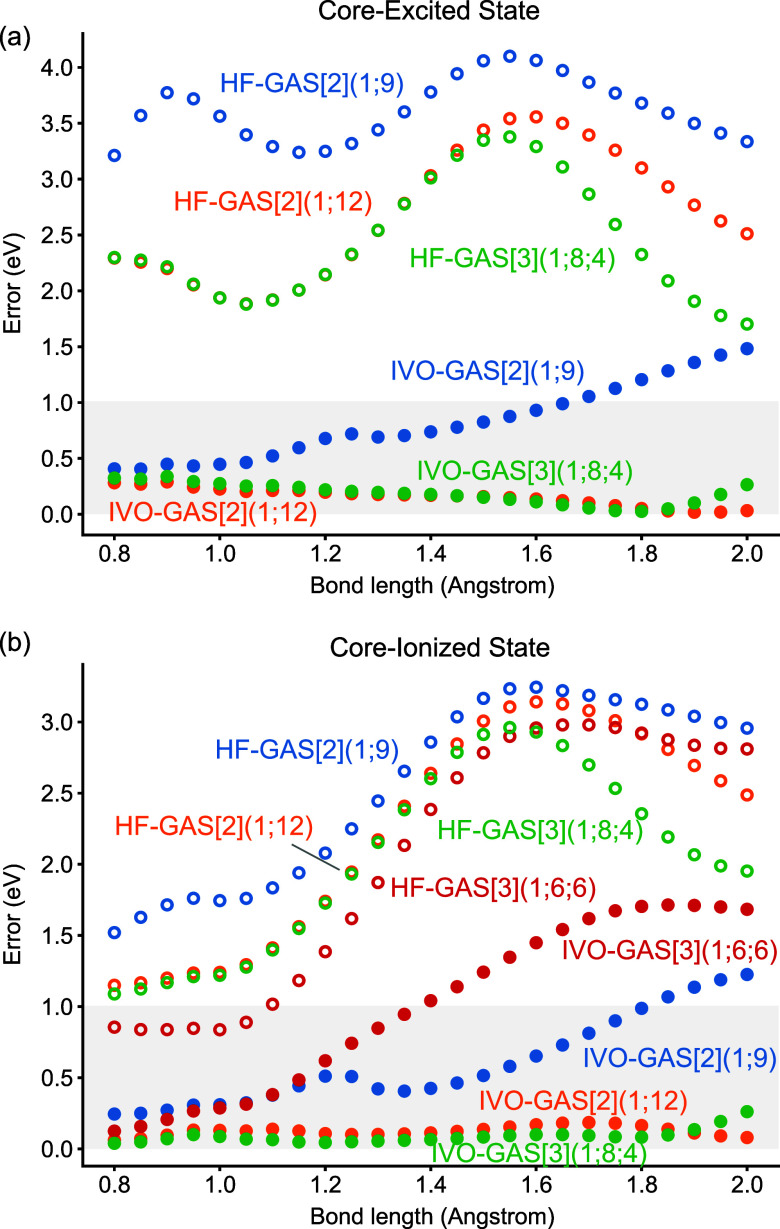
Relative errors in the potential energy surfaces of (a) the CO O 1s → π* core-excited state and (b) the CO O 1s core-ionized state computed using GASCI-DSRG-MRPT3 theory based on different orbitals and choices of active spaces compared to GASSCF-DSRG-MRPT3 results. The GASCI-DSRG-MRPT3 calculations use R­(O)­HF orbitals (open circles) and IVO orbitals (closed circles) under different GAS spaces and restrictions. Potential energy curves are calculated between 0.8 and 2.0 Å with a 0.05 Å spacing, using the cc-pCVQZ-DK basis and X2C one-electron scalar relativistic corrections.

In contrast, the IVO approach systematically improves the agreement with the GASSCF curves for both core-excited and core-ionized states. The IVO with a full core and valence active space shows relatively good agreement around the potential minimum; however, deviations from GASSCF at stretched geometries become significant. The IVO with a full core and valence active space, GAS[2](1;9), shows only a 0.5 eV error around the minimum at approximately 1.15 Å, but the deviations gradually increase to exceed 1.0–1.5 eV when the bond length reaches 2.0 Å. Expanding the active space, with or without occupation restrictions, leads to excellent agreement compared to the GASSCF result. As shown in Figure 2, expanding the active space to include three additional orbitals, whether using two subspaces [GAS(1;12)] or three subspaces [GAS(1;8;4)], reduces the overall error to less than 20 m*E*_h_ (ca. 0.54 eV) across all bond lengths when compared to the GASSCF-DSRG-MRPT3 results. Interestingly, we observe a peak in the error curve around 1.25 Å in both the core-excited and core-ionization curves. A detailed investigation attributes this feature to the rotation of two virtual orbitals generated from the IVO approach when the molecule stretches. As a result, it is not surprising that including these rotated orbitals in the active space leads to a much better agreement with the GASSCF results. Similarly, the GAS[3](1;6;6) active space (used in the previous section to calculate core-ionization energies at the equilibrium geometry) leads to a poorer agreement than the full core and valence active space GAS[2](1;9) at stretched bond lengths. This discrepancy is not surprising considering the extreme multideterminantal character of CO^+^ core-excited states wave functions, which have significant contributions from double excitations.^105,162^ These observations highlight the critical importance of active space selection in the IVO-GASCI approach.

In addition to the O K-edge of CO, we evaluated the PECs for the core-ionized and core-excited states of CO, N_2_, and HF. Table 2 lists the MAE and STD of all curves using both IVO and canonical orbitals with different active spaces, with GASSCF-DSRG-MRPT3 results serving as the reference. Our analysis indicates that, for all molecules in our test set, including higher-energy virtual orbitals (beyond the valence levels) is necessary to simulate the PECs accurately using the IVO-GASCI approach. Furthermore, we observe that IVO-GASCI[3] can yield results similar to IVO-GASCI[2] when using the same number of active orbitals while using only about one-tenth of the number of determinants. Aside from active space differences for ground state curves, the choice of orbitals does not significantly affect the calculated ground state energies, as the largest MAE with respect to GASSCF-DSRG-MRPT3 is 0.33 eV. However, we notice a significant orbital and element dependence in the error statistics for core-excited and core-ionized states. For the CO C 1s case, the IVO approach (using the largest determinant set) results in an MAE of 0.35 eV for core ionization and 0.17 eV for core excitation, which is worse than the 0.27 and 0.14 eV MAE obtained with canonical orbitals. Excluding the CO C 1s case, the largest MAE using IVO orbitals is 0.21 eV for the HF core-ionized state with a GAS[3](1;5;4) active space, whereas the smallest MAE with canonical orbitals is 0.32 eV for N_2_ with a GAS[3](2;7;5) active space. For other cases, the MAEs range from 1 to 3 eV. The standard deviation shows a similar trend; however, it is notably smaller for N_2_. Only in the CO O 1s and HF F 1s cases do IVO orbitals produce a significantly lower STD compared to canonical orbitals, underscoring the importance of the IVO approach in reproducing the shape of PECs.

**Table 2:** Error Statistics (in eV, MAE = Mean Absolute Error, STD = Standard Deviation) of the CO, N_2_, and HF Potential Energy Curves Computed Using GASCI-DSRG-MRPT3 with R­(O)­HF and IVO Orbitals and Various Active Space Partitions Compared to Reference GASSCF-DSRG-MRPT3^a^

Mol.	Orb.	active space	ground state			core ionization			core excitation		
			*N*_d_	MAE	STD	*N*_d_	MAE	STD	*N*_d_	MAE	STD
**C**O	R(O)HF	GAS[2](1;9)	1768	0.30	0.18	2840	1.00	1.16	1504	1.55	0.46
		GAS[3](1;8;4)	21,792	0.10	0.09	37,104	0.64	0.51	21,504	0.15	0.15
		GAS[2](1;12)	213,544	0.13	0.11	369,712	0.27	0.31	365,904	0.14	0.17
	IVO	GAS[2](1;9)	1768	0.22	0.22	2840	0.52	0.48	1504	0.51	0.24
		GAS[3](1;8;4)	21,792	0.05	0.06	37,104	0.33	0.37	21,504	0.18	0.20
		GAS[2](1;12)	213,544	0.07	0.10	369,712	0.35	0.38	365,904	0.17	0.19
**N**_2_	R(O)HF	GAS[2](2;8)	396	0.20	0.15	1256	0.91	0.85	784	1.15	0.22
		GAS[3](2;7;5)	6724	0.05	0.06	22,642	0.32	0.37	15,864	0.66	0.09
		GAS[2](2;12)	78,840	0.04	0.04	271,734	0.41	0.26	365,904	0.72	0.05
	IVO	GAS[2](2;8)	396	0.17	0.17	1256	1.07	0.58	784	0.63	0.27
		GAS[3](2;7;5)	6724	0.10	0.04	22,642	0.13	0.20	15,864	0.06	0.07
		GAS[2](2;12)	78,840	0.16	0.03	271,734	0.18	0.26	365,904	0.16	0.08
C**O**	R(O)HF	GAS[2](1;9)	1768	0.32	0.20	2840	2.55	0.64	1504	3.62	0.28
		GAS[3](1;8;4)	21,792	0.12	0.11	37,104	2.04	0.65	21,504	2.44	0.54
		GAS[2](1;12)	213,544	0.15	0.13	369,712	2.24	0.76	365,904	2.69	0.58
	IVO	GAS[2](1;9)	1768	0.21	0.24	2840	0.60	0.31	1504	0.82	0.34
		GAS[3](1;8;4)	21,792	0.12	0.09	37,104	0.09	0.05	21,504	0.19	0.09
		GAS[2](1;12)	213,544	0.14	0.06	369,712	0.13	0.03	365,904	0.16	0.08
H**F**	R(O)HF	GAS[2](1;5)	11	0.12	0.10	14	4.75	0.41	6	5.85	0.84
		GAS[3](1;5;4)	683	0.13	0.14	1093	1.51	0.99	706	1.67	0.90
		GAS[2](1;9)	4076	0.08	0.11	6716	1.78	0.77	8152	2.00	0.71
	IVO	GAS[2](1;5)	11	0.10	0.12	14	3.61	0.38	6	2.13	0.57
		GAS[3](1;5;4)	683	0.13	0.14	1093	0.07	0.08	706	0.21	0.12
		GAS[2](1;9)	4076	0.13	0.13	6716	0.06	0.08	8152	0.12	0.04

a The atom undergoing core ionization/excitation is in bold. Results cover bond distances between 0.8 and 2.0 Å with a 0.05 Å spacing, using the cc-pCVQZ-DK basis and a scalar X2C relativistic correction.

### 4.3 CORE65 and XABOOM Sets

Next, we employ the CORE65 ^32^ and XABOOM^149^ data sets of medium-size molecules to benchmark core-ionization and core-excitation energies computed with the IVO-GASCI-DSRG approach. The detailed transition energies are listed in Tables S9 and S10 in the Supporting Information. Table 3 reports IVO-GASCI[3]-DSRG-MRPT3 error statistics at the C, N, O, and F K-edges relative to experiment. IVO-GASCI[3]-DSRG-MRPT3 yields an overall MAE of 0.78 eV, trailing the more accurate GASSCF-DSRG-MRPT3 (MAE = 0.34 eV).^143^ As can be seen from Table 3, the element-specific error statistics indicate that the MAE varies significantly with the element type. The MAE at the C K-edge (0.34 eV) is lowest, rising to 0.74, 1.45, and 2.30 eV at the N, O, and F K-edges, respectively.

**Table 3:** Error Statistics (in eV, MAE = Mean Absolute Error, RMSE = Root Mean Square Error, STD = Standard Deviation) of IVO-GASCI[3]-DSRG Theories for the CORE65 Benchmark Set Using Experimental Values as Reference^a^

	*N*_T_	MAE	RMSE	STD
all	65	0.78	1.19	1.12
C 1s	30	0.34	0.47	0.42
N 1s	11	0.74	0.82	0.81
O 1s	21	1.45	1.86	1.29
F 1s	3	2.30	2.71	1.74

a For each element we list the number of transitions (*N*_T_). IVO-GASCI[3]-DSRG-MRPT3 results were computed using the cc-pVQZ basis.

The XABOOM data set on core-excitation energies shows worse performance compared to CORE65, as summarized in Table 4, with the overall MAE relative to experiment increasing to 1.00 eV. As with core-ionization, MAEs increase across the K-edges for individual elements: the C K-edge transitions show a relatively low MAE of 0.54 eV, which doubles to 1.10 eV for N K-edge and increases to 2.48 eV for O K-edge transitions. This trend reflects the increasing importance of orbital relaxation on core-ionized and core-excited states as the atomic number increases, an effect that is not fully captured by the IVO-GASCI approach. Moreover, a detailed investigation of the energies in Table S9 indicates that the transition energy errors for the O K-edge increase with molecular size, as the core-ionization energy error is small for the water molecule (0.33 eV) but becomes substantial for dimethyl ether (2.31 eV) and nitrobenzene (2.90 eV). For the XABOOM energies in Table S10, the adenine N K-edge and thymine O K-edge transitions show particularly large errors ranging from 2.3 to 4.3 eV. This explains the worse performance observed for the XABOOM set, which contains, on average, larger molecules compared to the CORE65 set, worsening the performance of the IVO approach in capturing orbital relaxation effects. These benchmarks show that while IVO-GASCI[3]-DSRG-MRPT3 serves as a good approximation for core transitions of small molecules, the insufficiency of IVO in capturing orbital relaxation effects leads to systematic errors for core energies in larger systems. One way to address this limitation is to increase the size of the GAS spaces. We will explore this strategy in the following section.

**Table 4:** Error Statistics (in eV, MAE = Mean Absolute Error, RMSE = Root Mean Square Error, STD = Standard Deviation) of IVO-GASCI[3]-DSRG Theories for the XABOOM Benchmark Set Using Experimental Values as Reference^a^

	*N*_T_	MAE	RMSE	STD
all	91	1.00	1.52	1.19
C 1s	57	0.54	0.70	0.53
N 1s	17	1.10	1.45	0.98
O 1s	17	2.48	2.79	1.71

a For each element we list the number of transitions (*N*_T_). IVO-GASCI[3]-DSRG-MRPT3 results were computed using the cc-pVQZ basis.

### 4.4 Thymine and Adenine Near-Edge X-ray Absorption Spectra

In this section, we employ the IVO-GASCI[3]-DSRG-MRPT3 method to simulate the X-ray absorption spectra of thymine and adenine, two essential nucleobases in biological systems. Notably, the spectra of these molecules exhibit multiple transitions, attributed to several near-degenerate core and π orbitals. While our original GAS-DSRG approach accurately reproduces the lowest core-excitation transitions from each atomic core, evaluating multiple transitions from the same core orbital is computationally demanding due to the rapidly increasing cost of orbital optimization for many states simultaneously. The IVO approach offers a more efficient alternative for simulations involving many excited states by avoiding the orbital optimization step.

Figures 3 and 4 show our IVO-GASCI[3]-DSRG-MRPT3 simulations of the C, N, and O K-edges of thymine and the C and N K-edges of adenine, alongside experimental data from Plekan et al.^163^ For comparison, we also include GASCI-DSRG-MRPT3 calculations using RHF orbitals. Detailed transition frequencies and oscillator strengths from IVO-GASCI[3]-DSRG-MRPT3 are listed in Tables S11–S15 of the Supporting Information, and the orbitals to which transitions are strong are plotted in Figures S3–S7 of the Supporting Information. The IVO-GASCI-DSRG-MRPT3 results agree qualitatively with the experimental data, requiring energy shifts ranging from 1.0 to 1.6 eV across different edges and molecules. These shifts are smaller than the 2.0 to 2.5 eV shifts used in the CVS-ADC(2) calculations used to assign the experimental spectra, and are comparable to those (1.0–1.6 eV) required for CVS-ADC(4) to simulate the XPS of adenine and thymine.^163^

**Figure 3:**
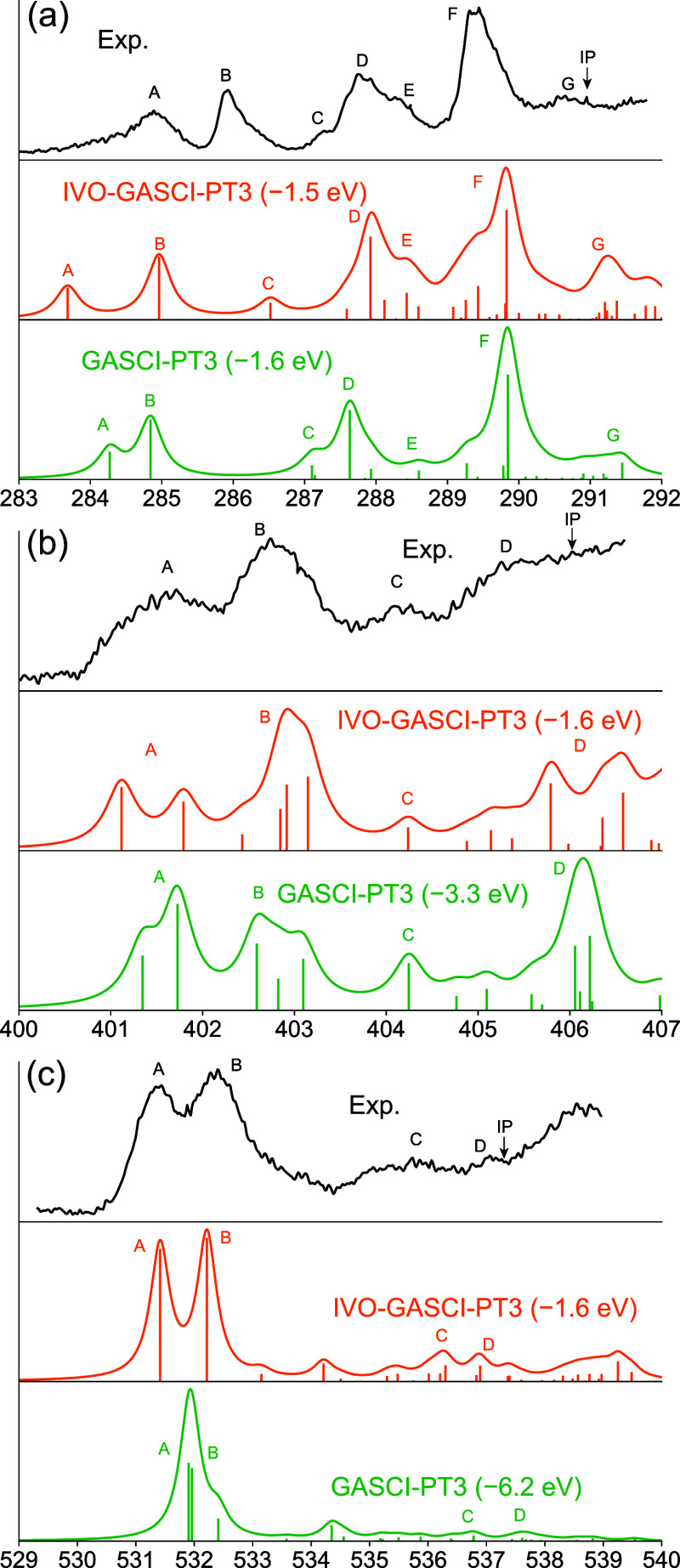
Comparison of experimental and theoretical X-ray absorption spectra for the (a) C K-edge, (b) N K-edge, and (c) O K-edge of thymine. Theoretical spectra are computed using IVO-GASCI-DSRG-PT3 and the cc-pVQZ basis set. GASCI-DSRG-MRPT3 results based on canonical RHF orbitals are also plotted. The computed spectra were convoluted with a Lorentzian function with full width at half-maximum (fwhm) equal to 0.3 eV. Experimental spectra are reproduced with permission from ref 163. Copyright 2008 Elsevier.

**Figure 4:**
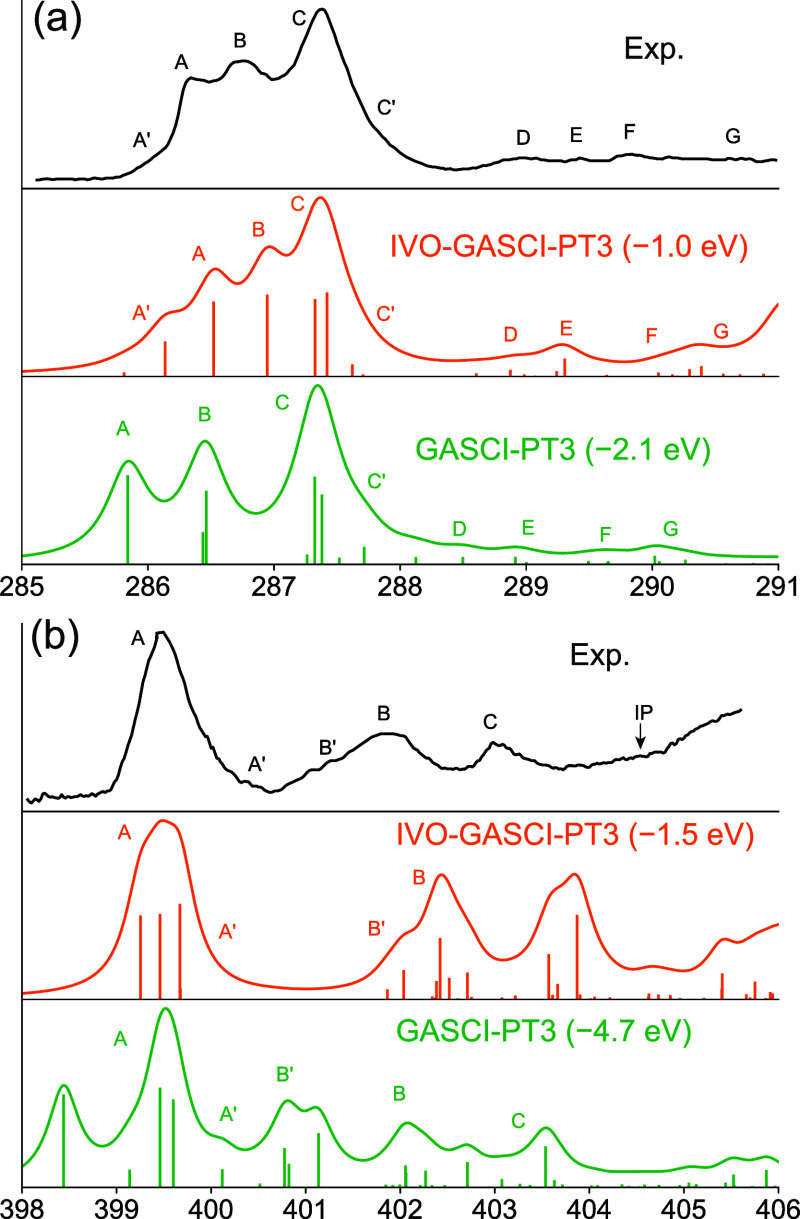
Comparison of experimental and theoretical X-ray absorption spectra for the (a) C K-edge and (b) N K-edge of adenine. Theoretical spectra are computed using IVO-GASCI-DSRG-PT3 and the cc-pVQZ basis set. GASCI-DSRG-MRPT3 results based on canonical RHF orbitals are also plotted. The computed spectra were convoluted with a Lorentzian function with full width at half-maximum (fwhm) equal to 0.3 eV. Experimental spectra are reproduced with permission from ref 163. Copyright 2008 Elsevier.

In terms of spectral features, we successfully reproduced most transitions of both nucleobases, though deviations in some intensities may be attributed to the fact that transition dipoles are evaluated at the GASCI level using the relaxed DSRG wave function, which may perform worse than those calculated at the GASSCF level.^141^ For example, the C K-edge spectrum of thymine reproduces the number of experimental peaks, with the experimental gaps between the lowest peaks (A–B = 1.0 eV, A–C = 2.4 eV) being reproduced fairly well by the IVO-GAS approach (A–B = 1.3 eV, A–C = 2.8 eV). The theoretical A–D gap (4.24 eV) is predicted to be ca. 1.3 eV larger than in the experiment (2.9 eV). In contrast, GASCI-DSRG-MRPT3 calculations based on RHF orbitals deviate significantly from experiment, requiring substantial shifts (1.6–6.2 eV) to achieve agreement. Furthermore, the gaps between the peaks show even larger deviations from experiment, with, for example, the C K-edge spectrum of thymine showing peak B shifted significantly to the red. Additionally, with RHF orbitals, this method failed to reproduce important spectral features, such as the singlet structure of the lowest N K-edge peak of adenine and the doublet structure of the lowest O K-edge peak of thymine. This highlights the potential of the IVO approach in modeling X-ray spectroscopies, as it effectively captures orbital relaxation effects in core-excited states, consistent with previous studies employing the IVO method for lower-resolution spectra simulations.^125^ Although methods such as CVS-ADC(2)^163^ and OCDFT^15^ can also provide similar qualitative agreement with the spectra of adenine and thymine—suggesting limited open-shell character in these transitions—our IVO-GASCI-DSRG approach remains a valuable tool for its ability to compute core spectroscopies of relatively large molecules with open-shell character, such as polyaromatic hydrocarbons (PAHs).

## 5 Conclusions

This study explores the IVO-GASCI-DSRG approach, which combines the Improved Virtual Orbitals (IVOs) with the Generalized Active Space (GAS) and multireference Driven Similarity Renormalization Group (MR-DSRG) to compute core-ionized and core-excited electronic states of molecules. In the IVO-GASCI-DSRG approach, core-ionized and core-excited states are first modeled by a generalized-active-space configuration interaction (GASCI), followed by a state-averaged treatment of dynamical electron correlation via MR-DSRG theory.

Our benchmark study on small molecules demonstrates the effectiveness of the IVO-GASCI-DSRG method in accurately simulating core-ionized and core-excited states. We first evaluate the accuracy of IVO-GASCI-DSRG theory using a full active space comprising core and valence orbitals (denoted as IVO-GASCI-DSRG[2]) for core-ionization and core-excitation energies in small molecules, comparing it with GASSCF-MR-LDSRG(2) theory.^143^ The IVO-GASCI[2]-DSRG shows an overall MAE of 1.48/0.62/0.48 eV for the DSRG-MRPT2, DSRG-MRPT3, and MR-LDSRG(2) levels of theory, which are significantly smaller than the corresponding 4.73/1.78/1.07 eV errors from calculations based on canonical R­(O)­HF orbitals. By expanding the active space and partitioning it into three subspaces (a RAS-like scheme denoted as GASCI[3]), the MAE is further reduced to 0.81/0.27/0.33 eV. The cost-effective IVO-GASCI[3]-DSRG-MRPT3 method outperforms GASSCF-DSRG-MRPT2 (0.29 eV MAE), which requires a more costly orbital optimization step. Larger active spaces can further improve the accuracy for specific molecules, such as F_2_ and CO_2_. For example, for F_2_, core-ionization/core-excitation errors decrease from 1.06/0.70 to 0.68/0.22 eV when the active space is increased from 14 to 16 orbitals; similarly, for the O K-edge of CO_2_, the errors decreases from 1.64/1.42 to 1.11/0.73 eV when the active space is expanded from 18 to 21 orbitals. Among all the approaches examined, the IVO-GASCI[3]-DSRG-MRPT3 offers the best balance between accuracy and efficiency.

We subsequently tested the IVO-GASCI[3]-DSRG-MRPT3 method to compute the potential energy curves (PECs) of the ground, core-ionized, and core-excited states of three representative first-row diatomic molecules (CO, N_2_, and HF). The PECs obtained with IVO-GASCI[3]-DSRG-MRPT3 exhibit MAEs of less than 0.33 eV compared to the GASSCF-DSRG-MRPT3 results. Our analysis shows that, when using the same active space, IVO-GASCI[3]-DSRG-MRPT3 achieves an accuracy comparable to that of IVO-GASCI[2]-DSRG-MRPT3, yet requiring only about one-tenth the number of determinants. Lastly, we employed the IVO-GASCI[3]-DSRG-MRPT3 method to simulate the X-ray absorption spectra of thymine and adenine, which involve multiple transitions.^163^ These simulations agree well with experimental data after applying small energy shifts (1.0–1.6 eV), comparable to those used at the ADC(4) levels.^163^ The IVO approach successfully captures key spectral features that GASCI-DSRG-MRPT3 based on R­(O)­HF orbitals fails to reproduce. Thus, we believe it will be a valuable tool for simulating the XAS and XPS of large molecules, particularly those with open-shell electronic structure.

This study demonstrates that the IVO-GASCI-DSRG approach can accurately predict core ionization and excitation energies; however, it also highlights several challenges. At present, dipole moments within the IVO-GASCI-DSRG framework are calculated only at the IVO-GASCI level, neglecting the effect of dynamical correlation. Incorporating the effects of dynamical correlation may be essential to accurately predict XAS transition dipole moments and intensities. Additionally, the current IVO approach is based on Hartree–Fock orbitals and targets core-ionized states. This suggests that there might be room for improving the description of systems with open-shell character and core-excited states. This is indeed demonstrated by our benchmarks using the CORE65 and XABOOM sets, which reveal that IVO excitation energies degrade with increasing system size. One potential solution is to develop IVOs based on a minimal complete active space self-consistent field (CASSCF) or approximate orbital-optimized methods,^164–168^ benefiting from the advantages of IVOs and orbital optimization. Such an approach would also better handle the orbital near degeneracies in more complex situations not considered in this work, such as conical intersections.

Further theoretical advancements could also involve applying the IVO-GASCI-DSRG method to efficiently evaluate XAS for transient species in their valence-excited states.

## Supplementary Material



## Data Availability

The data that supports the findings of this study are available within the article and its Supporting Information.

## Notes

Additional details and calculations: analysis of the absolute IVO-GASCI-DSRG energies; active space used for IVO-GASCI-DSRG calculation; error statistics and distribution for the core-ionization and core-excited energies of small molecules, compared to experimental data; energies of IVO-GASCI-DSRG on the CORE65 and XABOOM datasets; and transition frequencies and intensities for the X-ray absorption spectrum of thymine and adenine, computed using IVO-GASCI-DSRG-MRPT3, along with the final orbitals associated with transitions of significant intensity (PDF)
