# Supplementary material for: An Improved Virtual Orbital Driven Similarity Renormalization Group Approach for Core-Ionized and Core-Excited States
Source: J Chem Theory Comput. 2025 Jul 11;21(14):6834–48. doi: 10.1021/acs.jctc.5c00457 (PMC12288017; doi:10.1021/acs.jctc.5c00457)
Supplement: Supplementary file 1 [file ct5c00457_si_001.pdf]

# Supporting Information: An Improved Virtual Orbital Driven Similarity Renormalization Group Approach for Core-Ionized and Core-Excited States

Meng Huang† and Francesco A. Evangelista*,‡

†*State Key Laboratory of Precision and Intelligent Chemistry, University of Science and Technology of China, Hefei, Anhui 230026, China*

‡*Department of Chemistry and Cherry Emerson Center for Scientific Computation, Emory University, Atlanta, Georgia, 30322, U.S.A.*

E-mail: francesco.evangelista@emory.edu

# S1 Absolute IVO-GASCI-DSRG Energies

We tested the accuracy of IVO-GASCI-DSRG methods by computing the absolute core-ionization and core-excitation energies in small molecules and comparing them to the GASSCF-DSRG results obtained with the same level of DSRG theory. Table S1 shows the Mean Absolute Error (MAE) and Standard Deviation (STD) values for all the ground, core-ionized, and core-excited state energies, using both IVO and HF orbitals (denoted with GASCI without any prefix). The GASCI calculations, based on Hartree-Fock orbitals and without dynamic correlation, yield a significant Mean Absolute Error (MAE) of 16.04/13.69 eV for core-ionization/core-excitation states compared to the GASSCF energies, due to the neglect of core relaxation effects. The IVO-GASCI approach partially accounts for these dynamic correlations, reducing the MAE to a lower 8.53/6.02 eV. However, these errors remain significant without any dynamic correlation treatment. Using canonical orbitals, DSRG-MRPT2 theories reduce the error to 4.68/4.39 eV. More accurate dynamical correlation treatments, such as DSRG-MRPT3 and LDSRG(2), further reduce the error to 1.42/2.25 eV and 0.98/1.34 eV, respectively. Combining IVO and DSRG theories unsurprisingly yields the best agreement, decreasing the error to an acceptable ∼0.5 eV compared to the GASSCF-DSRG results for the accurate DSRG-MRPT3 and MR-LDSRG(2) theories. The standard errors follow a similar trend as the absolute errors, indicating systematic improvement.

In terms of error for different states, we observed that the error in the ground state has relatively less dependence on the orbitals. Interestingly, the IVO orbitals optimized for core-excited/core-ionized states result in a significantly smaller MAE (1.51 eV) compared to those optimized under HF orbitals (2.81 eV). These errors are further reduced by the inclusion of dynamic correlation via DSRG theories, where the MAEs are around 0.10 eV for both HF and IVO orbitals with any DSRG theories. These small errors align with our previous calculations on the transition dipole moment of XAS, where we can approximate the ground state using a GASCI calculation based on the core-excited state-optimized GASSCF orbitals.[1]

Table S1: Error statistics (in eV, MAE = Mean absolute error, STD = Standard deviation) of absolute GASCI-DSRG energies, compared to the GASSCF-DSRG results under the same level of DSRG theories. All results were computed using the cc-pCVQZ-DK basis and X2C for relativistic correction.

| | Ground State | | Core Ionization | | Core Excitation | |
|---|---|---|---|---|---|---|
| | MAE | STD | MAE | STD | MAE | STD |
| GASCI | 2.81 | 0.96 | 16.04 | 2.49 | 13.69 | 3.55 |
| GASCI-DSRG-MRPT2 | 0.10 | 0.08 | 4.68 | 3.66 | 4.39 | 3.68 |
| GASCI-DSRG-MRPT3 | 0.12 | 0.06 | 1.42 | 1.66 | 2.25 | 1.76 |
| GASCI-MR-LDSRG(2) | 0.10 | 0.06 | 0.98 | 0.57 | 1.34 | 0.69 |
| IVO-GASCI | 1.51 | 0.68 | 8.53 | 2.32 | 6.02 | 2.32 |
| IVO-GASCI-DSRG-MRPT2 | 0.13 | 0.10 | 1.77 | 1.97 | 0.96 | 1.27 |
| IVO-DSRG-MRPT3 | 0.07 | 0.05 | 0.56 | 0.72 | 0.66 | 0.69 |
| IVO-MR-LDSRG(2) | 0.05 | 0.04 | 0.41 | 0.27 | 0.58 | 0.31 |

Table S2: The active space used for IVO-GASCI[2]-DSRG and IVO-GASCI[3]-DSRG calculations on the core-ionized state of each molecule.

| Molecule | GASCI[2] | GASCI[3] |
|---|---|---|
| $H\mathbf{F}$ | 1o,1e; 5o, 4e | 1o,1e, 1e; 4o, 4e, 6e; 2o, 0e, 2e |
| $\mathbf{C}O$ | 2o,3e; 9o, 6e | 1o,1e, 1e; 6o, 8e, 10e; 6o, 0e, 2e |
| $C\mathbf{O}$ | 2o,3e; 9o, 6e | 1o,1e, 1e; 6o, 8e, 10e; 6o, 0e, 2e |
| $\mathbf{N}_2$ | 2o,3e; 8o, 6e | 2o,3e, 3e; 5o, 6e, 8e; 7o, 0e, 2e |
| $\mathbf{F}_2$ | 3o,5e; 8o, 8e | 2o,3e, 3e; 7o, 10e, 12e; 5o, 0e, 2e |
| $H_2\mathbf{O}$ | 1o,1e; 6o, 4e | 1o,1e, 1e; 4o, 2e, 4e; 2o, 0e, 2e |
| $\mathbf{C}_2H_4$ | 3o,5e; 12o, 6e | 2o,3e, 3e; 6o, 8e, 10e; 10o, 0e, 2e |
| $\mathbf{C}_2H_2$ | 2o,3e; 10o, 6e | 2o,3e, 3e; 5o, 6e, 8e; 9o, 0e, 2e |
| $\mathbf{C}H_4$ | 1o,1e; 8o, 4e | 1o,1e, 1e; 4o, 4e, 6e; 7o, 0e, 2e |
| $CH_2\mathbf{O}$ | 1o,1e; 11o, 10e | 1o,1e, 1e; 7o, 10e, 12e; 7o, 0e, 2e |
| $\mathbf{C}H_2O$ | 1o,1e; 11o, 10e | 1o,1e, 1e; 7o, 10e, 12e; 7o, 0e, 2e |
| $C\mathbf{O}_2$ | 1o,1e; 14o, 16e | 2o,3e, 1e; 10o, 16e, 18e; 8o, 0e, 2e |
| $\mathbf{C}O_2$ | 2o,3e; 13o, 14e | 1o,1e, 3e; 9o, 14e, 16e; 8o, 0e, 2e |
| $NN\mathbf{O}$ | 2o,3e; 14o, 14e | 1o,1e, 1e; 12o, 16e, 18e; 5o, 0e, 2e |
| $N\mathbf{N}O$ | 2o,3e; 14o, 14e | 1o,1e, 1e; 12o, 16e, 18e; 5o, 0e, 2e |
| $\mathbf{N}NO$ | 2o,3e; 14o, 14e | 1o,1e, 1e; 12o, 16e, 18e; 5o, 0e, 2e |
| $\mathbf{N}H_3$ | 1o,1e; 7o, 4e | 1o,1e, 1e; 4o, 4e, 6e; 6o, 0e, 2e |
| $H\mathbf{C}N$ | 1o,1e; 10o, 8e | 1o,1e, 1e; 6o, 8e, 10e; 7o, 0e, 2e |
| $HC\mathbf{N}$ | 2o,3e; 10o, 6e | 1o,1e, 1e; 6o, 8e, 10e; 7o, 0e, 2e |
| $CH_3\mathbf{O}H$ | 1o,1e; 13o, 12e | 1o,1e, 1e; 8o, 12e, 14e; 7o, 0e, 2e |
| $\mathbf{C}H_3OH$ | 1o,1e; 13o, 12e | 1o,1e, 1e; 8o, 12e, 14e; 7o, 0e, 2e |

Table S3: The active space used for IVO-GASCI[2]-DSRG and IVO-GASCI[3]-DSRG calculations on the core-excited state of small molecules.

| Molecule | GASCI[2] | GASCI[3] |
|---|---|---|
| H**F** | 1o,1e; 5o, 5e | 1o,1e, 1e; 5o, 5e, 7e; 4o, 0e, 2e |
| **C**O | 2o,3e; 9o, 7e | 1o,1e, 1e; 8o, 9e, 11e; 4o, 0e, 2e |
| C**O** | 2o,3e; 9o, 7e | 1o,1e, 1e; 8o, 9e, 11e; 4o, 0e, 2e |
| $\mathbf{N}_2$ | 2o,3e; 8o, 7e | 2o,3e, 3e; 7o, 7e, 9e; 5o, 0e, 2e |
| $\mathbf{F}_2$ | 3o,5e; 8o, 9e | 2o,3e, 3e; 8o, 11e, 13e; 4o, 0e, 2e |
| $H_2\mathbf{O}$ | 1o,1e; 6o, 5e | 1o,1e, 1e; 5o, 5e, 7e; 4o, 0e, 2e |
| $\mathbf{C}_2H_4$ | 3o,5e; 12o, 7e | 2o,3e, 3e; 7o, 9e, 11e; 9o, 0e, 2e |
| $\mathbf{C}_2H_2$ | 2o,3e; 10o, 7e | 2o,3e, 3e; 7o, 7e, 9e; 7o, 0e, 2e |
| $\mathbf{C}H_4$ | 1o,1e; 8o, 5e | 1o,1e, 1e; 5o, 5e, 7e; 6o, 0e, 2e |
| $CH_2\mathbf{O}$ | 1o,1e; 11o, 11e | 1o,1e, 1e; 8o, 11e, 13e; 6o, 0e, 2e |
| $\mathbf{C}H_2O$ | 1o,1e; 11o, 11e | 1o,1e, 1e; 8o, 11e, 13e; 6o, 0e, 2e |
| $C\mathbf{O}_2$ | 1o,1e; 14o, 17e | 2o,3e, 3e; 11o, 15e, 17e; 6o, 0e, 2e |
| $\mathbf{C}O_2$ | 2o,3e; 13o, 15e | 1o,1e, 1e; 12o, 17, 19e; 6o, 0e, 2e |
| NN**O** | 2o,3e; 14o, 15e | 1o,1e, 1e; 12o, 17e, 19e; 5o, 0e, 2e |
| N**N**O | 2o,3e; 14o, 15e | 1o,1e, 1e; 12o, 17e, 19e; 5o, 0e, 2e |
| **N**NO | 2o,3e; 14o, 15e | 1o,1e, 1e; 12o, 17e, 19e; 5o, 0e, 2e |
| $\mathbf{N}H_3$ | 1o,1e; 7o, 5e | 1o,1e, 1e; 5o, 5e, 7e; 5o, 0e, 2e |
| H**C**N | 1o,1e; 10o, 9e | 1o,1e, 1e; 8o, 9e, 11e; 5o, 0e, 2e |
| HC**N** | 2o,3e; 10o, 7e | 1o,1e, 1e; 8o, 9e, 11e; 5o, 0e, 2e |
| $CH_3\mathbf{O}H$ | 1o,1e; 13o, 13e | 1o,1e, 1e; 9o, 13e, 15e; 6o, 0e, 2e |
| $\mathbf{C}H_3OH$ | 1o,1e; 13o, 13e | 1o,1e, 1e; 9o, 13e, 15e; 6o, 0e, 2e |

Table S4: GASCI[2]-DSRG core-ionization energies of small molecules (in eV), calculated using R(O)HF and IVO orbitals under different levels of DSRG theory. All results were computed using the cc-pCVQZ-DK basis and X2C for relativistic correction.

| Molecule | R(O)HF Orbitals | | | IVO Orbitals | | |
|---|---|---|---|---|---|---|
| | PT2 | PT3 | LDSRG(2) | PT2 | PT3 | LDSRG(2) |
| $H\mathbf{F}$ | 682.38 | 699.67 | 694.49 | 686.69 | 697.32 | 694.70 |
| $\mathbf{C}O$ | 295.16 | 296.19 | 298.11 | 296.13 | 296.26 | 296.76 |
| $C\mathbf{O}$ | 536.68 | 544.48 | 543.13 | 540.49 | 543.13 | 543.22 |
| $\mathbf{N}_2$(g) | 408.17 | 410.28 | 410.95 | 408.68 | 410.73 | 410.90 |
| $\mathbf{F}_2$(g) | 690.07 | 700.06 | 697.27 | 691.83 | 699.44 | 697.27 |
| $H_2\mathbf{O}$ | 529.31 | 544.06 | 540.66 | 536.23 | 541.26 | 540.76 |
| $\mathbf{C}_2H_4$(g) | 290.43 | 291.58 | 292.29 | 289.30 | 290.72 | 291.73 |
| $\mathbf{C}_2H_2$(g) | 290.28 | 291.34 | 292.91 | 291.07 | 292.18 | 292.65 |
| $\mathbf{C}H_4$ | 287.28 | 290.81 | 292.14 | 290.54 | 290.97 | 291.18 |
| $CH_2\mathbf{O}$ | 531.14 | 542.60 | 540.79 | 536.99 | 540.31 | 540.18 |
| $\mathbf{C}H_2O$ | 292.09 | 294.53 | 296.09 | 294.33 | 294.57 | 294.86 |
| $C\mathbf{O}_2$ | 536.63 | 543.76 | 542.17 | 538.05 | 543.46 | 542.28 |
| $\mathbf{C}O_2$ | 296.28 | 297.84 | 299.40 | 297.77 | 297.61 | 298.00 |
| $NN\mathbf{O}$ | 535.10 | 543.78 | 542.08 | 540.27 | 542.08 | 541.96 |
| $N\mathbf{N}O$ | 410.35 | 413.08 | 413.23 | 412.43 | 412.83 | 413.14 |
| $\mathbf{N}NO$ | 405.66 | 409.14 | 409.73 | 408.33 | 408.85 | 409.14 |
| $\mathbf{N}H_3$ | 404.50 | 406.13 | 406.42 | 404.50 | 406.13 | 406.42 |
| $H\mathbf{C}N$ | 402.78 | 407.68 | 407.71 | 406.11 | 407.06 | 407.40 |
| $HC\mathbf{N}$ | 290.75 | 293.93 | 295.43 | 293.19 | 293.58 | 294.16 |
| $CH_3\mathbf{O}H$ | 527.60 | 544.09 | 540.20 | 537.74 | 539.67 | 539.59 |
| $\mathbf{C}H_3OH$ | 288.89 | 292.64 | 294.05 | 292.19 | 292.61 | 292.77 |

Table S5: GASCI[3]-DSRG core-ionization energies of small molecules (in eV), calculated using R(O)HF and IVO orbitals under different levels of DSRG theory. All results were computed using the cc-pCVQZ-DK basis and X2C for relativistic correction.

| Molecule | R(O)HF Orbitals | | | IVO Orbitals | | |
|---|---|---|---|---|---|---|
| | PT2 | PT3 | LDSRG(2) | PT2 | PT3 | LDSRG(2) |
| H**F** | 689.08 | 694.71 | 694.60 | 693.36 | 694.71 | 694.64 |
| **C**O | 295.60 | 295.35 | 296.76 | 296.50 | 296.25 | 296.74 |
| C**O** | 535.88 | 543.79 | 543.34 | 541.76 | 543.12 | 543.22 |
| $\mathbf{N}_2$(g) | 409.08 | 410.20 | 410.84 | 410.20 | 410.63 | 410.90 |
| $\mathbf{F}_2$(g) | 694.18 | 697.83 | 697.36 | 694.64 | 698.30 | 697.50 |
| $H_2\mathbf{O}$ | 532.79 | 541.36 | 540.63 | 538.10 | 540.43 | 540.68 |
| $\mathbf{C}_2H_4$(g) | 289.50 | 290.31 | 291.83 | 290.61 | 291.08 | 291.55 |
| $\mathbf{C}_2H_2$(g) | 290.39 | 290.61 | 292.30 | 291.64 | 291.31 | 292.26 |
| $\mathbf{C}H_4$ | 287.60 | 290.34 | 292.25 | 290.40 | 290.63 | 291.20 |
| $CH_2\mathbf{O}$ | 531.38 | 541.34 | 540.97 | 538.63 | 539.83 | 540.03 |
| $\mathbf{C}H_2O$ | 292.43 | 293.49 | 295.39 | 294.40 | 294.38 | 295.02 |
| $C\mathbf{O}_2$ | 537.34 | 543.19 | 542.24 | 538.73 | 543.36 | 542.34 |
| $\mathbf{C}O_2$ | 297.00 | 297.15 | 298.52 | 298.08 | 297.96 | 298.52 |
| NN**O** | 536.14 | 543.05 | 542.09 | 541.02 | 541.81 | 541.87 |
| N**N**O | 410.45 | 412.83 | 413.16 | 412.45 | 412.87 | 413.03 |
| **N**NO | 406.36 | 408.59 | 409.41 | 408.48 | 408.83 | 408.95 |
| $\mathbf{N}H_3$ | 400.41 | 405.66 | 406.49 | 404.98 | 405.63 | 405.99 |
| H**C**N | 402.74 | 406.89 | 408.13 | 406.78 | 407.22 | 407.59 |
| HC**N** | 291.91 | 292.59 | 294.19 | 293.76 | 293.65 | 294.18 |
| $CH_3\mathbf{O}H$ | 528.19 | 543.45 | 540.28 | 538.10 | 539.47 | 539.62 |
| $\mathbf{C}H_3OH$ | 289.06 | 292.33 | 294.22 | 292.09 | 292.35 | 292.93 |

Table S6: GASCI[2]-DSRG core-excitation energies of small molecules (in eV), calculated using R(O)HF and IVO orbitals under different levels of DSRG theory. All results were computed using the cc-pCVQZ-DK basis and X2C for relativistic correction.

| Molecule | R(O)HF Orbitals | | | IVO Orbitals | | |
|---|---|---|---|---|---|---|
| | PT2 | PT3 | LDSRG(2) | PT2 | PT3 | LDSRG(2) |
| $\mathrm{H}\mathbf{F}$ | 683.02 | 688.95 | 688.65 | 687.53 | 688.74 | 688.70 |
| $\mathbf{C}\mathrm{O}$ | 286.86 | 287.42 | 288.16 | 287.86 | 287.63 | 287.76 |
| $\mathrm{C}\mathbf{O}$ | 528.46 | 536.69 | 535.22 | 533.86 | 535.06 | 535.17 |
| $\mathbf{N}_2$(g) | 400.13 | 402.18 | 402.35 | 400.81 | 401.57 | 401.50 |
| $\mathbf{F}_2$(g) | 681.04 | 684.17 | 683.50 | 681.60 | 684.44 | 683.62 |
| $\mathrm{H}_2\mathbf{O}$ | 527.73 | 536.67 | 535.55 | 533.33 | 535.43 | 535.55 |
| $\mathbf{C}_2\mathrm{H}_4$(g) | 284.25 | 285.58 | 286.63 | 284.97 | 285.59 | 285.69 |
| $\mathbf{C}_2\mathrm{H}_2$(g) | 285.81 | 287.11 | 287.89 | 286.05 | 286.58 | 286.66 |
| $\mathbf{C}\mathrm{H}_4$ | 284.54 | 287.42 | 289.15 | 287.68 | 288.06 | 288.55 |
| $\mathrm{CH}_2\mathbf{O}$ | 523.87 | 534.18 | 532.56 | 531.08 | 531.91 | 532.18 |
| $\mathbf{C}\mathrm{H}_2\mathrm{O}$ | 284.63 | 285.94 | 286.95 | 286.15 | 286.34 | 286.57 |
| $\mathrm{C}\mathbf{O}_2$ | 531.77 | 538.11 | 536.67 | 533.12 | 537.24 | 536.54 |
| $\mathbf{C}\mathrm{O}_2$ | 290.27 | 291.13 | 291.75 | 291.24 | 291.28 | 291.40 |
| $\mathrm{NN}\mathbf{O}$ | 529.46 | 536.76 | 536.00 | 534.64 | 535.47 | 535.72 |
| $\mathrm{N}\mathbf{N}\mathrm{O}$ | 399.10 | 401.88 | 402.19 | 401.22 | 401.61 | 401.74 |
| $\mathbf{N}\mathrm{NO}$ | 402.70 | 405.90 | 405.80 | 404.93 | 405.33 | 405.44 |
| $\mathbf{N}\mathrm{H}_3$ | 396.38 | 401.88 | 402.47 | 401.00 | 401.74 | 402.08 |
| $\mathrm{H}\mathbf{C}\mathrm{N}$ | 396.47 | 401.62 | 401.48 | 400.18 | 400.62 | 400.84 |
| $\mathrm{HC}\mathbf{N}$ | 285.49 | 286.89 | 287.84 | 286.65 | 286.86 | 287.09 |
| $\mathrm{CH}_3\mathbf{O}\mathrm{H}$ | 524.38 | 539.99 | 536.54 | 534.37 | 535.78 | 535.92 |
| $\mathbf{C}\mathrm{H}_3\mathrm{OH}$ | 285.91 | 289.23 | 291.01 | 288.81 | 289.18 | 289.67 |

Table S7: GASCI[3]-DSRG core-excitation energies of small molecules (in eV), calculated using R(O)HF and IVO orbitals under different levels of DSRG theory. All results were computed using the cc-pCVQZ-DK basis and X2C for relativistic correction.

| Molecule | R(O)HF Orbitals | | | IVO Orbitals | | |
|---|---|---|---|---|---|---|
| | PT2 | PT3 | LDSRG(2) | PT2 | PT3 | LDSRG(2) |
| H**F** | 676.71 | 694.78 | 689.27 | 683.34 | 691.57 | 689.39 |
| **C**O | 287.56 | 289.37 | 289.61 | 287.86 | 287.93 | 288.45 |
| C**O** | 528.87 | 537.84 | 536.07 | 533.45 | 535.28 | 535.50 |
| $\mathbf{N}_2$(g) | 399.25 | 402.43 | 402.46 | 400.23 | 401.98 | 401.68 |
| $\mathbf{F}_2$(g) | 677.78 | 687.13 | 683.97 | 679.91 | 685.57 | 683.55 |
| $H_2\mathbf{O}$ | 524.47 | 539.76 | 536.07 | 532.67 | 536.68 | 536.32 |
| $\mathbf{C}_2H_4$(g) | 284.37 | 285.91 | 286.92 | 284.56 | 285.62 | 285.71 |
| $\mathbf{C}_2H_2$(g) | 285.71 | 287.21 | 288.37 | 286.00 | 286.86 | 287.08 |
| $\mathbf{C}H_4$ | 284.20 | 287.84 | 286.89 | 287.38 | 287.90 | 288.19 |
| $CH_2\mathbf{O}$ | 523.28 | 535.71 | 533.29 | 530.35 | 532.27 | 532.58 |
| $\mathbf{C}H_2O$ | 284.71 | 287.53 | 288.45 | 286.60 | 286.78 | 286.97 |
| $C\mathbf{O}_2$ | 531.21 | 539.46 | 537.41 | 532.53 | 538.28 | 536.68 |
| $\mathbf{C}O_2$ | 290.83 | 293.09 | 293.97 | 291.38 | 291.41 | 291.60 |
| NN**O** | 528.22 | 537.82 | 536.13 | 534.74 | 536.00 | 536.31 |
| N**N**O | 398.36 | 402.49 | 402.47 | 401.66 | 401.99 | 402.24 |
| **N**NO | 402.49 | 406.10 | 405.82 | 405.38 | 405.51 | 405.75 |
| $\mathbf{N}H_3$ | 394.59 | 403.78 | 401.78 | 400.95 | 402.37 | 402.27 |
| H**C**N | 396.42 | 402.43 | 401.94 | 400.31 | 400.85 | 401.19 |
| HC**N** | 285.18 | 288.67 | 289.57 | 287.11 | 287.42 | 287.69 |
| $CH_3\mathbf{O}H$ | 523.81 | 540.65 | 536.46 | 534.00 | 535.78 | 535.82 |
| $\mathbf{C}H_3OH$ | 285.76 | 289.54 | 290.93 | 288.87 | 289.33 | 289.57 |

Table S8: Core-ionization and core-excitation energies of small molecules. Error statistics (in eV, MAE = Mean absolute error, STD = Standard deviation) of GAS-DSRG theories based on different set of references compared to experimental results. Results are reported for both a small active space with 2 GAS spaces (GASCI[2]) and a larger one with 3 GAS spaces (GASCI[3]) when using HF and IVO orbitals. For GASSCF, only the 2 GAS spaces results are reported. All results were computed using the cc-pCVQZ-DK basis and X2C for relativistic correction.

| | Overall | | Core Ionization | | Core Excitation | |
|---|---|---|---|---|---|---|
| | MAE | STD | MAE | STD | MAE | STD |
| GASCI[2]-DSRG-MRPT2 | 4.20 | 3.45 | 4.47 | 3.55 | 3.92 | 3.44 |
| GASCI[2]-DSRG-MRPT3 | 2.29 | 1.97 | 1.55 | 1.75 | 3.04 | 1.89 |
| GASCI[2]-MR-LDSRG(2) | 1.58 | 0.79 | 1.14 | 0.50 | 2.01 | 0.81 |
| GASCI[3]-DSRG-MRPT2 | 3.37 | 2.68 | 3.71 | 2.77 | 3.03 | 2.62 |
| GASCI[3]-DSRG-MRPT3 | 1.28 | 1.42 | 0.94 | 1.28 | 1.61 | 1.33 |
| GASCI[3]-MR-LDSRG(2) | 1.24 | 0.60 | 0.94 | 0.35 | 1.55 | 0.57 |
| IVO-GASCI[2]-DSRG-MRPT2 | 1.26 | 1.75 | 1.55 | 1.92 | 0.92 | 1.32 |
| IVO-GASCI[2]-DSRG-MRPT3 | 1.13 | 1.04 | 0.75 | 0.91 | 1.50 | 0.97 |
| IVO-GASCI[2]-MR-LDSRG(2) | 1.01 | 0.54 | 0.65 | 0.29 | 1.37 | 0.38 |
| IVO-GASCI[3]-DSRG-MRPT2 | 0.60 | 0.81 | 0.67 | 0.82 | 0.47 | 0.66 |
| IVO-GASCI[3]-DSRG-MRPT3 | 0.72 | 0.65 | 0.45 | 0.54 | 0.98 | 0.52 |
| IVO-GASCI[3]-MR-LDSRG(2) | 0.86 | 0.47 | 0.64 | 0.24 | 1.08 | 0.42 |
| GASSCF-DSRG-MRPT2 | 0.33 | 0.39 | 0.16 | 0.15 | 0.45 | 0.40 |
| GASSCF-DSRG-MRPT3 | 0.56 | 0.52 | 0.23 | 0.17 | 0.88 | 0.48 |
| GASSCF-MR-LDSRG(2) | 0.55 | 0.42 | 0.26 | 0.12 | 0.82 | 0.31 |

Table S9: The core-ionized energies of molecules in the CORE65 data set[2] from experiment and GAS-DSRG theories. Results were calculated with different reference orbitals and DSRG-MRPT3 levels of theory [IVO-PT3 = IVO-GASCI[3]-DSRG-MRPT3, GASSCF-PT3 = GASSCF-DSRG-MRPT3] using the cc-pVQZ basis. The energies are in eV.

| Name | Formula | Core level | Exp. | IVO-PT3 | GASSCF-PT3 |
|---|---|---|---|---|---|
| methane | $CH_4$ | C1s | 290.84 | 290.15 | 290.53 |
| ethane | $C_2H_6$ | C1s | 290.71 | 290.24 | 290.45 |
| ethene | $C_2H_4$ | C1s | 290.82 | 290.55 | 290.69 |
| ethyne | $C_2H_2$ | C1s | 291.25 | 291.27 | 291.11 |
| carbon monoxide | CO | O1s | 542.10 | 542.28 | 541.89 |
| carbon monoxide | CO | C1s | 296.23 | 295.76 | 295.74 |
| carbon dioxide | $CO_2$ | O1s | 541.32 | 542.59 | 541.00 |
| carbon dioxide | $CO_2$ | C1s | 297.70 | 297.50 | 297.28 |
| tetrafluoromethane | $CF_4$ | F1s | 695.20 | 697.81 | 694.94 |
| tetrafluoromethane | $CF_4$ | C1s | 301.90 | 301.73 | 301.65 |
| fluoromethane | $CH_3F$ | F1s | 692.40 | 692.82 | 692.16 |
| fluoromethane | $CH_3F$ | C1s | 293.56 | 292.95 | 293.21 |
| trifluoromethane | $CHF_3$ | F1s | 694.10 | 697.97 | 694.50 |
| trifluoromethane | $CHF_3$ | C1s | 299.16 | 298.89 | 298.87 |
| methanol | $CH_3OH$ | O1s | 538.88 | 538.64 | 538.55 |
| methanol | $CH_3OH$ | C1s | 292.30 | 291.88 | 292.20 |
| formaldehyde | $CH_2O$ | O1s | 539.33 | 539.02 | 538.95 |
| formaldehyde | $CH_2O$ | C1s | 294.38 | 293.91 | 294.17 |
| dimethyl ether | $CH_3OCH_3$ | O1s | 538.36 | 540.22 | 537.91 |
| dimethyl ether | $CH_3OCH_3$ | C1s | 292.17 | 291.48 | 291.90 |
| formic acid | HCOOH | O1s (OH) | 540.69 | 542.17 | 540.21 |
| formic acid | HCOOH | O1s (C=O) | 539.02 | 539.16 | 538.48 |
| formic acid | HCOOH | C1s | 295.75 | 295.22 | 295.49 |
| acetone | $(CH_3)_2CO$ | O1s | 537.73 | 539.33 | 537.61 |
| acetone | $(CH_3)_2CO$ | C1s (C=O) | 293.88 | 294.01 | 293.44 |
| acetone | $(CH_3)_2CO$ | C1s ($CH_3$) | 291.23 | 290.49 | 291.09 |
| methyl formate | $HCO_2CH_3$ | O1s ($OCH_3$) | 539.64 | 543.16 | 539.81 |
| methyl formate | $HCO_2CH_3$ | O1s (C=O) | 538.24 | 539.92 | 538.11 |
| acetic acid | $CH_3COOH$ | O1s (OH) | 540.10 | 543.77 | 539.46 |
| acetic acid | $CH_3COOH$ | O1s (C=O) | 538.31 | 539.18 | 538.11 |
| acetic acid | $CH_3COOH$ | C1s (COOH) | 295.35 | 296.00 | 295.27 |
| acetic acid | $CH_3COOH$ | C1s ($CH_3$) | 291.55 | 291.84 | 291.26 |
| water | $H_2O$ | O1s | 539.70 | 539.37 | 539.26 |
| ozone | $O_3$ | O1s middle | 546.44 | 545.71 | 545.64 |
| ozone | $O_3$ | O1s terminal | 541.75 | 542.23 | 541.35 |
| oxygen | $O_2$ | O1s weaker | 544.20 | 544.23 | 543.78 |
| oxygen | $O_2$ | O1s stronger | 543.10 | 543.04 | 542.86 |
| nitrogen | $N_2$ | N1s | 409.93 | 409.95 | 409.46 |
| ammonia | $NH_3$ | N1s | 405.52 | 405.05 | 405.05 |
| hydrogen cyanide | HCN | N1s | 406.80 | 406.61 | 405.92 |

| | | | | | |
|---|---|---|---|---|---|
| hydrogen cyanide | HCN | C1s | 293.50 | 293.13 | 293.46 |
| acetonitrile | $CH_3CN$ | N1s | 405.58 | 405.03 | 405.05 |
| acetonitrile | $CH_3CN$ | C1s ($CH_3$) | 292.88 | 292.50 | 292.21 |
| acetonitrile | $CH_3CN$ | C1s (CN) | 292.60 | 292.26 | 292.29 |
| glycine | $C_2H_5NO_2$ | O1s (OH) | 540.20 | 543.28 | 539.83 |
| glycine | $C_2H_5NO_2$ | O1s (C=O) | 538.40 | 539.15 | 537.78 |
| glycine | $C_2H_5NO_2$ | N1s | 405.40 | 406.43 | 405.15 |
| glycine | $C_2H_5NO_2$ | C1s (COOH) | 295.20 | 295.52 | 295.06 |
| glycine | $C_2H_5NO_2$ | C1s ($CH_2$) | 292.30 | 292.76 | 291.96 |
| pyridine | $C_5H_5N$ | N1s | 404.82 | 405.84 | 404.16 |
| pyrrole | $C_4H_4NH$ | N1s | 406.18 | 406.71 | 405.73 |
| aniline | $C_6H_5NH_2$ | N1s | 405.31 | 407.33 | 404.90 |
| urea | $CO(NH_2)_2$ | O1s | 537.19 | 538.38 | 536.61 |
| urea | $CO(NH_2)_2$ | N1s | 406.09 | 406.36 | 406.05 |
| urea | $CO(NH_2)_2$ | C1s | 294.84 | 295.59 | 294.63 |
| methylamine | $CH_3NH_2$ | N1s | 405.17 | 404.78 | 404.69 |
| nitrobenzene | $C_6H_5NO_2$ | O1s | 538.63 | 540.92 | 538.03 |
| nitrobenzene | $C_6H_5NO_2$ | N1s | 411.60 | 411.25 | 411.38 |
| nitrobenzene | $C_6H_5NO_2$ | C1s ($C_1$) | 292.08 | 291.95 | 292.43 |
| nitrobenzene | $C_6H_5NO_2$ | C1s ($C_{2-4}$) | 291.13 | 290.61 | 290.89 |
| benzene | $C_6H_6$ | C1s | 290.38 | 290.11 | 290.02 |
| phenylacetylene | $C_8H_6$ | C1s ($C_3$) | 290.88 | 290.85 | 291.17 |
| phenylacetylene | $C_8H_6$ | C1s ($C_2$) | 290.55 | 290.66 | 290.46 |
| phenylacetylene | $C_8H_6$ | C1s ($C_{4-6}$) | 290.16 | 290.29 | 290.43 |
| phenylacetylene | $C_8H_6$ | C1s ($C_1$) | 289.75 | 290.17 | 289.67 |

Table S10: The core-excited energies of molecules in the XABOOM data set[3] from experiment and GAS-DSRG theories. Results were calculated with different reference orbitals and DSRG-MRPT3 levels of theory [IVO-PT3 = IVO-GASCI[3]-DSRG-MRPT3, GASSCF-PT3 = GASSCF-DSRG-MRPT3] using the cc-pVQZ basis. The energies are in eV.

| Molecule | Exp. | IVO-PT3 | GASSCF-PT3 |
|---|---|---|---|
| | **C K-edge** | | |
| HCN | 286.4 | 286.25 | 286.45 |
| HNC | | 286.54 | 286.98 |
| CO | 287.4 | 287.07 | 287.16 |
| $C_2H_4$ | 284.7 | 285.03 | 285.14 |
| HCHO | 285.6 | 285.74 | 285.95 |
| CH3CN | 286.9 | 287.10 | 286.71 |
| $CH_2CHF$ | 285.0 | 285.30 | 285.50 |
| | 287.1 | 287.36 | 287.60 |
| Acetamide($CH_3CONH_2$) | | 288.40 | 288.78 |
| Acetic Acid($CH_3COOH$) | 288.7 | 288.81 | 288.57 |
| CHF=CHF | 285.4 | 285.84 | 285.80 |
| | 289.6 | 289.94 | 290.12 |
| $CF_2O$ | 290.9 | 290.85 | 291.17 |
| Cyclopentadiene | 284.7 | 284.58 | 284.75 |
| | 284.7 | 285.01 | 285.14 |
| Furan | 285.8 | 287.46 | 286.00 |
| | 286.6 | 287.51 | 286.90 |
| Imidazole | 286.7 | 286.98 | 286.73 |
| | 286.7 | 287.31 | 286.99 |
| | 286.7 | 287.64 | 287.53 |
| Oxazole | | 286.56 | 286.43 |
| | | 287.13 | 286.95 |
| | | 287.59 | 287.60 |
| Glyoxylic Acid | | 285.69 | 285.92 |
| | | 287.77 | 288.01 |
| Methyl Acetate | | 288.51 | 288.52 |
| Benzene | 285.2 | 285.23 | 285.32 |
| Pyridine | 284.9 | 284.98 | 285.25 |
| | 284.9 | 285.07 | 285.26 |
| | 285.5 | 285.62 | 285.78 |
| Pyridazine | 285.5 | 284.97 | 285.02 |
| | 286.2 | 285.61 | 285.80 |
| s-Triazine | 285.9 | 285.92 | 286.36 |
| Trifluoroethene | 287.6 | 287.78 | 288.08 |
| | 289.7 | 289.87 | 290.20 |
| Dimethyl Carbonate | 290.3 | 290.39 | 290.47 |
| Trifluoroacetonitrile | 286.7 | 286.46 | 286.24 |
| Dichloroethene | | 285.25 | 285.27 |
| | | 288.06 | 287.79 |

| | | | |
|---|---|---|---|
| Benzoquinone | | 283.27 | 283.50 |
| | | 283.89 | 284.02 |
| | | 285.33 | 285.48 |
| Cytosine | 285.0 | 285.38 | 284.62 |
| | 286.0 | 287.18 | 285.90 |
| | 287.4 | 288.65 | 286.99 |
| | 289.3 | 290.37 | 288.84 |
| Uracil | 284.7 | 284.89 | 284.99 |
| | 285.9 | 286.42 | 286.40 |
| | 288.0 | 288.03 | 288.21 |
| | 289.3 | 289.75 | 289.90 |
| Cyclohexadione | 286.6 | 286.89 | 286.77 |
| Difluorbenzene | 285.3 | 285.54 | 285.40 |
| | 287.6 | 287.47 | 287.63 |
| Thymine | 284.9 | 285.44 | 284.45 |
| | 285.9 | 286.89 | 285.93 |
| | 287.8 | 289.20 | 287.47 |
| | 289.4 | 290.65 | 289.21 |
| Naphtalene | 284.6 | 284.95 | 284.75 |
| | 284.8 | 285.03 | 284.98 |
| | 285.5 | 285.73 | 285.70 |
| Adenine | 286.4 | 287.42 | 286.26 |
| | 286.4 | 287.39 | 286.23 |
| | 286.8 | 287.90 | 286.63 |
| | 287.4 | 288.48 | 287.15 |
| | 287.4 | 288.55 | 287.33 |
| Guanine | 286.1 | 286.49 | 285.93 |
| | 286.9 | 287.59 | 286.76 |
| | 286.9 | 288.44 | 287.19 |
| | 288.2 | 289.54 | 288.05 |
| | 288.7 | 289.55 | 288.30 |
| Perfluorodiene | | 287.40 | 287.85 |
| | | 289.24 | 289.52 |
| **N K-edge** | | | |
| HCN | 399.7 | 399.94 | 399.87 |
| HNC | | 400.77 | 400.79 |
| HNO | | 398.82 | 398.78 |
| $CH_3CN$ | 399.9 | 399.94 | 399.63 |
| $N_2O$ | 401.1 | 401.14 | 401.20 |
| | 404.7 | 404.82 | 404.72 |
| $HN_2F$ | | 399.19 | 399.46 |
| | | 401.34 | 401.52 |
| $CH_3NO_2$ | 403.9 | 405.80 | 404.30 |
| Imidazole | 399.9 | 400.76 | 399.69 |
| Oxazole | 399.9 | 400.40 | 399.48 |
| Pyridine | 398.8 | 399.45 | 398.65 |

| | | | |
|---|---|---|---|
| Pyridazine | 399.0 | 400.04 | 399.89 |
| s-Triazene | 398.9 | 399.60 | 399.44 |
| Trifluoroacetonitrile | 399.8 | 399.75 | 399.63 |
| Cytosine | 399.1 | 400.09 | 398.05 |
| Adenine | 399.5 | 401.38 | 399.02 |
| Adenine | 399.5 | 401.67 | 399.10 |
| Adenine | 399.5 | 402.36 | 398.94 |
| Guanine | 399.6 | 401.18 | 399.24 |
| Guanine | 399.6 | 402.62 | 399.76 |
| **O K-edge** | | | |
| CO | 533.6 | 534.14 | 533.83 |
| HCHO | 530.8 | 531.04 | 530.79 |
| HNO | | 530.32 | 530.23 |
| $N_2O$ | 535.1 | 534.55 | 534.50 |
| $O_3$ | 529.4 | 530.41 | 530.24 |
| | 535.0 | 535.37 | 535.38 |
| Acetamide($CH_3CONH_2$) | | 534.11 | 531.71 |
| Acetic Acid($CH_3COOH$) | 532.0 | 534.41 | 531.68 |
| $CH_3NO_2$ | 531.7 | 534.39 | 531.95 |
| | 531.7 | 534.55 | 531.96 |
| $CF_2O$ | 532.7 | 535.52 | 532.66 |
| Glyoxylic Acid | | 532.25 | 530.46 |
| | | 532.71 | 530.67 |
| Methyl Acetate | | 535.16 | 531.84 |
| Dimethyl Carbonate | 532.9 | 535.16 | 532.84 |
| Benzoquinone | | 532.32 | 530.37 |
| Cytosine | 532.0 | 536.04 | 531.52 |
| Uracil | 531.4 | 533.31 | 531.00 |
| | 532.4 | 535.88 | 532.17 |
| Cyclohexadione | 531.1 | 537.12 | 531.55 |
| Thymine | 531.4 | 534.13 | 530.56 |
| | 532.3 | 536.03 | 531.75 |
| Guanine | 531.9 | 536.38 | 531.17 |

Table S11: C K-edge XAS transition energies and relative intensities (with respect to the strongest transition) of thymine, computed using IVO-GASCI-DSRG-PT3 with the cc-pVQZ basis set. The experimental frequencies[4] are also listed. The theoretical frequencies are shifted by −1.5 eV to align with the experimental results. The transitions with significant relative intensities ($f^{\mathrm{rel}} > 0.10$) are colored blue.

| Transition | Theory | | Experiment | |
|---|---|---|---|---|
| | $\omega$ (eV) | $f^{\mathrm{rel}}$ | Maximum | $\omega$ (eV) |
| $C_8$ 1s $\rightarrow$ $\pi_1^*$ | 283.68 | 0.305 | A | 284.9 |
| $C_7$ 1s $\rightarrow$ $\pi_1^*$ | 284.96 | 0.602 | B | 285.9 |
| $C_9$ 1s $\rightarrow$ $\pi_2^*$ | 286.50 | 0.000 | | |
| $C_8$ 1s $\rightarrow$ $\pi_2^*$ | 286.52 | 0.163 | C | 287.3 |
| $C_8$ 1s $\rightarrow D_3/D_4$ | 286.98 | 0.003 | | |
| $C_9$ 1s $\rightarrow D_2$ | 287.59 | 0.102 | | |
| $C_6$ 1s $\rightarrow$ $\pi_1^*$ | 287.92 | 0.791 | D | 287.8 |
| $C_8$ 1s $\rightarrow D_3/D_5$ | 287.94 | 0.013 | | |
| $C_7$ 1s $\rightarrow D_1$ | 288.12 | 0.189 | | |
| $C_8$ 1s $\rightarrow D_5$ | 288.28 | 0.012 | | |
| $C_7$ 1s $\rightarrow$ $\pi_2^*$ | 288.39 | 0.002 | | |
| $C_9$ 1s $\rightarrow D_1$ | 288.43 | 0.255 | E | 288.4 |
| $C_9$ 1s $\rightarrow$ $\pi_1^*/\pi_3$ | 288.59 | 0.126 | | |
| $C_9$ 1s $\rightarrow D_5/D_3$ | 289.08 | 0.124 | | |
| $C_8$ 1s $\rightarrow D_9/D_5$ | 289.19 | 0.023 | | |
| $C_8$ 1s $\rightarrow$ $\pi_3^*$ | 289.26 | 0.189 | | |
| $C_5$ 1s $\rightarrow$ $\pi_1^*$ | 289.28 | 1.000 | F | 289.4 |
| $C_7$ 1s $\rightarrow D_4$ | 289.29 | 0.010 | | |
| $C_9$ 1s $\rightarrow D_1/D_5$ | 289.43 | 0.320 | | |
| $C_8$ 1s $\rightarrow D_8$ | 289.59 | 0.027 | | |
| $C_7$ 1s $\rightarrow D_3$ | 289.69 | 0.051 | | |
| $C_6$ 1s $\rightarrow$ $\pi_2^*$ + HOMO$\rightarrow \pi_1$ | 289.81 | 0.154 | | |
| $C_9$ 1s $\rightarrow D_5/D_4$ | 289.98 | 0.009 | | |
| $C_8$ 1s $\rightarrow D_6$ | 290.00 | 0.065 | | |
| $C_7$ 1s $\rightarrow$ $\pi_1^*$ + HOMO$-2 \rightarrow \pi_1$ | 290.08 | 0.000 | | |
| $C_7$ 1s $\rightarrow D_1$ + HOMO$\rightarrow D_1$ | 290.28 | 0.056 | | |
| $C_8$ 1s $\rightarrow D_7$ | 290.37 | 0.058 | | |
| $C_5$ 1s $\rightarrow$ $\pi_2^*$ | 290.47 | 0.063 | | |
| $C_9$ 1s $\rightarrow D_6$ | 290.57 | 0.050 | | |
| $C_8$ 1s $\rightarrow D_{14}$ | 290.57 | 0.033 | | |
| $C_8$ 1s $\rightarrow D_9$ | 290.58 | 0.000 | | |

Table S11: C K-edge XAS transition energies and relative intensities (with respect to the strongest transition) of thymine, computed using IVO-GASCI-DSRG-PT3 with the cc-pVQZ basis set. The experimental frequencies[4] are also listed. The theoretical frequencies are shifted by −1.5 eV to align with the experimental results. The transitions with significant relative intensities ($f^{\mathrm{rel}} > 0.10$) are colored blue.

| Transition | Theory | | Experiment | |
|---|---|---|---|---|
| | $\omega$ (eV) | $f^{\mathrm{rel}}$ | Maximum | $\omega$ (eV) |
| $C_7$ 1s $\rightarrow D_8$ | 290.72 | 0.009 | | |
| $C_6$ 1s $\rightarrow \pi_1^*$ + HOMO−2 $\rightarrow \pi_1^*$ | 290.75 | 0.000 | | |
| $C_7$ 1s $\rightarrow \pi_4^*$ | 290.84 | 0.011 | | |
| $C_7$ 1s $\rightarrow D_7$ | 291.03 | 0.015 | | |
| $C_9$ 1s $\rightarrow D_8$ | 291.09 | 0.027 | | |
| $C_7$ 1s $\rightarrow \pi_1^*$ + HOMO−1 $\rightarrow$ 34 | 291.12 | 0.069 | | |
| $C_9$ 1s $\rightarrow D_9$ | 291.21 | 0.169 | G | 290.7 |
| $C_8$ 1s $\rightarrow \pi_2^*$ + HOMO−1 $\rightarrow \pi_1^*$ | 291.23 | 0.000 | | |
| $C_8$ 1s $\rightarrow D_{10}$ | 291.23 | 0.088 | | |
| $C_8$ 1s $\rightarrow D_{13}$ | 291.37 | 0.182 | | |
| $C_7$ 1s $\rightarrow D_5/D_7$ | 291.42 | 0.002 | | |
| $C_6$ 1s $\rightarrow \pi_1^*$ + HOMO $\rightarrow \pi_1^*$ | 291.62 | 0.055 | | |
| $C_9$ 1s $\rightarrow \pi_2^*$ + HOMO−1 $\rightarrow D_2^*$ | 291.73 | 0.000 | | |

Table S12: N K-edge XAS transition energies and relative intensities (with respect to the strongest transition) of thymine, computed using IVO-GASCI-DSRG-PT3 with the cc-pVQZ basis set. The experimental frequencies[4] are also listed. The theoretical frequencies are shifted by −1.6 eV to align with the experimental results. The transitions with significant relative intensities ($f^{\mathrm{rel}} > 0.10$) are colored blue.

| Transition | Theory | | Experiment | |
|---|---|---|---|---|
| | $\omega$ (eV) | $f^{\mathrm{rel}}$ | Maximum | $\omega$ (eV) |
| $N_4$ 1s $\rightarrow$ $\pi_1^*$ | 401.12 | 0.650 | | |
| $N_3$ 1s $\rightarrow$ $\pi_1^*$ | 401.79 | 0.499 | A | 401.7 |
| $N_4$ 1s $\rightarrow$ $\pi_2^*$ | 402.43 | 0.166 | | |
| $N_3$ 1s $\rightarrow$ $\pi_2^*$ | 402.85 | 0.425 | | |
| $N_3$ 1s $\rightarrow$ $D_1$ | 402.92 | 0.673 | B | 402.7 |
| $N_4$ 1s $\rightarrow$ $D_1$ | 403.15 | 0.756 | | |
| $N_4$ 1s $\rightarrow$ $D_5$ | 404.24 | 0.237 | C | 404.1 |
| $N_4$ 1s $\rightarrow$ $D_4$ | 404.88 | 0.098 | | |
| $N_3$ 1s $\rightarrow$ $D_3/D_4$ | 405.14 | 0.209 | | |
| $N_3$ 1s $\rightarrow$ $D_4$ | 405.28 | 0.002 | | |
| $N_4$ 1s $\rightarrow$ $D_6$ | 405.37 | 0.126 | | |
| $N_4$ 1s $\rightarrow$ $\pi_3^*$ | 405.79 | 0.689 | D | 405.5 |
| $N_3$ 1s $\rightarrow$ $D_7$ | 405.98 | 0.070 | | |
| $N_4$ 1s $\rightarrow$ $D_8$ | 406.34 | 0.048 | | |
| $N_3$ 1s $\rightarrow$ $D_8$ | 406.36 | 0.337 | | |
| $N_3$ 1s $\rightarrow$ $\pi_4^*$ | 406.58 | 0.591 | | |
| $N_4$ 1s $\rightarrow$ $D_7$ | 406.89 | 0.108 | | |
| $N_3$ 1s $\rightarrow$ $D_6$ | 406.97 | 0.074 | | |
| $N_4$ 1s $\rightarrow$ $D_{11}$ | 407.10 | 0.332 | | |
| HOMO $\rightarrow$ $\pi_1^*$ + $N_4$ 1s $\rightarrow$ $\pi_5^*$ | 407.14 | 0.014 | | |
| $N_4$ 1s $\rightarrow$ $D_3$ | 407.32 | 0.419 | | |
| $N_4$ 1s $\rightarrow$ $\pi_5^*$ + HOMO $\rightarrow$ $\pi_1^*$ | 407.39 | 0.015 | | |
| $N_3$ 1s $\rightarrow$ $D_{10}$ | 407.56 | 0.218 | | |
| $N_4$ 1s $\rightarrow$ $D_{11}$ | 407.68 | 0.228 | | |
| $N_4$ 1s $\rightarrow$ $\pi_6^*$ | 407.76 | 0.121 | | |
| $N_3$ 1s $\rightarrow$ $\pi_3^*$ | 407.86 | 0.007 | | |
| $N_3$ 1s $\rightarrow$ $D_{11}$ | 408.00 | 0.096 | | |
| $N_3$ 1s $\rightarrow$ $D_2$ | 408.06 | 1.000 | | |
| $N_3$ 1s $\rightarrow$ $D_9/D_2$ | 408.24 | 0.479 | | |
| $N_3$ 1s $\rightarrow$ $\pi_1^*/\pi_2^*$ + HOMO $\rightarrow$ $D_1$ | 408.59 | 0.011 | | |

Table S13: O K-edge XAS transition energies and relative intensities (with respect to the strongest transition) of thymine, computed using IVO-GASCI-DSRG-PT3 with the cc-pVQZ basis set. The experimental frequencies[4] are also listed. The theoretical frequencies are shifted by −1.6 eV to align with the experimental results. The transitions with significant relative intensities ($f^{\text{rel}} > 0.10$) are colored blue.

| Transition | Theory | | Experiment | |
|---|---|---|---|---|
| | $\omega$ (eV) | $f^{\text{rel}}$ | Maximum | $\omega$ (eV) |
| $O_2$ 1s $\rightarrow \pi_1^*$ | 531.42 | 0.925 | A | 531.4 |
| $O_1$ 1s $\rightarrow \pi_1^*$ | 532.22 | 1.000 | B | 532.3 |
| $O_1$ 1s $\rightarrow \pi_2^*$ | 533.15 | 0.052 | | |
| $O_2$ 1s $\rightarrow \pi_2^*$ | 534.21 | 0.124 | | |
| $O_1$ 1s $\rightarrow D_2$ | 534.51 | 0.019 | | |
| $O_2$ 1s $\rightarrow D_4$ | 535.30 | 0.038 | | |
| $O_1$ 1s $\rightarrow D_3$ | 535.48 | 0.054 | | |
| $O_2$ 1s $\rightarrow D_5/D_4$ | 535.75 | 0.008 | | |
| $O_2$ 1s $\rightarrow D_3$ | 536.01 | 0.055 | | |
| $O_1$ 1s $\rightarrow D_3/D_1$ | 536.21 | 0.056 | | |
| $O_1$ 1s $\rightarrow D_6/D_2$ | 536.30 | 0.113 | C | 535.7 |
| $O_2$ 1s $\rightarrow D_6$ | 536.83 | 0.044 | | |
| $O_2$ 1s $\rightarrow \pi_4^*$ | 536.89 | 0.111 | D | 537.1 |
| $O_1$ 1s $\rightarrow D_8$ | 537.37 | 0.037 | | |
| $O_1$ 1s $\rightarrow D_7$ | 537.40 | 0.041 | | |
| $O_2$ 1s $\rightarrow D_8$ | 537.60 | 0.012 | | |
| $O_1$ 1s $\rightarrow \pi_3^*$ | 537.65 | 0.006 | | |
| $O_2$ 1s $\rightarrow \pi_3^*$ | 537.95 | 0.008 | | |
| $O_2$ 1s $\rightarrow D_5/D_4$ | 538.16 | 0.012 | | |
| $O_2$ 1s $\rightarrow D_9$ | 538.31 | 0.040 | | |
| $O_1$ 1s $\rightarrow D_9$ | 538.47 | 0.020 | | |
| $O_1$ 1s $\rightarrow \pi_4^*$ | 538.57 | 0.048 | | |
| $O_2$ 1s $\rightarrow \pi_1^*$ + HOMO $\rightarrow \pi_1^*$ | 538.69 | 0.006 | | |
| $O_1$ 1s $\rightarrow D_{10}$ | 538.76 | 0.053 | | |
| $O_1$ 1s $\rightarrow D_{11}$ | 538.92 | 0.017 | | |
| $O_2$ 1s $\rightarrow D_{11}$ | 538.97 | 0.052 | | |
| $O_2$ 1s $\rightarrow \pi_5^*/\pi_6^*$ | 539.19 | 0.000 | | |
| $O_1$ 1s $\rightarrow \pi_5^*$ | 539.25 | 0.140 | | |
| $O_2$ 1s $\rightarrow D_{10}$ | 539.48 | 0.066 | | |
| $O_1$ 1s $\rightarrow D_8/D_{11}/D_{12}$ | 539.89 | 0.003 | | |

Table S14: N K-edge XAS transition energies and relative intensities (with respect to the strongest transition) of adenine, computed using IVO-GASCI-DSRG-PT3 with the cc-pVQZ basis set. The experimental frequencies[4] are also listed. The theoretical frequencies are shifted by −1.6 eV to align with the experimental results.

| Transition | Theory | | Experiment | |
|---|---|---|---|---|
| | $\omega$ (eV) | $f^{\text{rel}}$ | Maximum | $\omega$ (eV) |
| $N_4 \rightarrow \pi_1^*$ | 399.25 | 0.907 | | |
| $N_3 \rightarrow \pi_1^*$ | 399.39 | 1.000 | A | 399.5 |
| $N_5 \rightarrow \pi_1^*$ | 399.46 | 0.921 | | |
| $N_5 \rightarrow \pi_2^*$ | 399.68 | 0.119 | A′ | 400.4 |
| $N_4 \rightarrow \pi_2^*$ | 400.65 | 0.006 | | |
| $N_3 \rightarrow \pi_2^*$ | 401.44 | 0.131 | | |
| $N_2 \rightarrow \pi_1^*$ | 402.04 | 0.317 | B′ | 401.0–401.3 |
| $N_3 \rightarrow \pi_3^*$ | 402.08 | 0.234 | | |
| $N_5 \rightarrow \pi_3^*$ | 402.34 | 0.031 | | |
| $N_3 \rightarrow D_4$ | 402.35 | 0.006 | | |
| $N_5 \rightarrow D_1$ | 402.36 | 0.018 | | |
| $N_4 \rightarrow \pi_3^*$ | 402.38 | 0.198 | | |
| $N_1 \rightarrow \pi_1^*$ | 402.42 | 0.662 | B | 401.9 |
| $N_4 \rightarrow D_2$ | 402.61 | 0.013 | | |
| $N_2 \rightarrow D_1$ | 402.71 | 0.290 | | |
| $N_5 \rightarrow D_7$ | 402.75 | 0.028 | | |
| $N_4 \rightarrow D_7/\pi_1^*$ | 403.08 | 0.020 | | |
| $N_2 \rightarrow \pi_2^*$ | 403.22 | 0.043 | | |
| $N_3 \rightarrow \pi_3^*$ | 403.28 | 0.013 | | |
| $N_5 \rightarrow D_4$ | 403.28 | 0.001 | | |
| $N_3 \rightarrow D_3$ | 403.52 | 0.051 | | |
| $N_1 \rightarrow D_1$ | 403.57 | 0.489 | C | 403.0 |
| $N_4 \rightarrow D_4$ | 403.60 | 0.012 | | |
| $N_5 \rightarrow D_5$ | 403.61 | 0.048 | | |
| $N_1 \rightarrow \pi_2^*$ | 403.66 | 0.168 | | |
| $N_2 \rightarrow D_2$ | 403.87 | 0.912 | | |
| $N_4 \rightarrow D_6$ | 404.01 | 0.011 | | |
| $N_5 \rightarrow D_9$ | 404.06 | 0.029 | | |
| $N_3 \rightarrow D_6$ | 404.13 | 0.004 | | |
| $N_5 \rightarrow D_9/D_6$ | 404.22 | 0.022 | | |
| $N_4 \rightarrow D_7/D_8$ | 404.32 | 0.007 | | |
| $N_4 \rightarrow D_{10}$ | 404.62 | 0.030 | | |

Table S14: N K-edge XAS transition energies and relative intensities (with respect to the strongest transition) of adenine, computed using IVO-GASCI-DSRG-PT3 with the cc-pVQZ basis set. The experimental frequencies[4] are also listed. The theoretical frequencies are shifted by −1.6 eV to align with the experimental results.

| Transition | Theory | | Experiment | |
|---|---|---|---|---|
| | $\omega$ (eV) | $f^{\mathrm{rel}}$ | Maximum | $\omega$ (eV) |
| $N_1 \rightarrow \pi_3^*$ | 404.63 | 0.064 | | |
| $N_2 \rightarrow \pi_3^*$ | 404.73 | 0.054 | | |
| $N_3 \rightarrow D_7$ | 404.85 | 0.002 | | |
| $N_5 \rightarrow \pi_1^*$ + HOMO$\rightarrow \pi_1^*$ | 404.86 | 0.046 | | |
| $N_2 \rightarrow D_7$ | 404.96 | 0.016 | | |
| $N_3 \rightarrow D_9$ | 405.20 | 0.052 | | |
| $N_5 \rightarrow D_2$ | 405.21 | 0.018 | | |
| $N_3 \rightarrow \pi_1^*$ + HOMO$\rightarrow \pi_1^*$ | 405.38 | 0.012 | | |
| $N_4 \rightarrow \pi_1^*$ + HOMO$\rightarrow \pi_1^*$ | 405.40 | 0.115 | | |
| $N_5 \rightarrow \pi_5^*$ | 405.41 | 0.281 | | |
| $N_3 \rightarrow D_5/D_9$ | 405.47 | 0.058 | | |
| $N_2 \rightarrow D_5$ | 405.70 | 0.022 | | |
| $N_4 \rightarrow \pi_5^*$ | 405.75 | 0.192 | | |
| $N_1 \rightarrow D_3$ | 405.91 | 0.081 | | |
| $N_4 \rightarrow D_{11}$ | 405.94 | 0.064 | | |
| $N_3 \rightarrow \pi_1^*$ + HOMO−1 $\rightarrow \pi_1^*$ | 405.95 | 0.010 | | |
| $N_5 \rightarrow D_{12}$ | 406.04 | 0.152 | | |
| $N_1 \rightarrow D_4$ | 406.09 | 0.049 | | |
| $N_3 \rightarrow \pi_5^*/\pi_4^*$ | 406.19 | 0.017 | | |
| $N_3 \rightarrow D_{11}$ | 406.22 | 0.018 | | |
| $N_4 \rightarrow D_{12}$ | 406.23 | 0.163 | | |
| $N_2 \rightarrow D_8$ | 406.24 | 0.001 | | |
| $N_2 \rightarrow D_{10}$ | 406.33 | 0.121 | | |
| $N_5 \rightarrow \pi_2^*$ + HOMO$\rightarrow \pi_1^*$ | 406.36 | 0.353 | | |
| $N_4 \rightarrow D_8$ | 406.39 | 0.058 | | |
| $N_5 \rightarrow D_{12}/D_{10}$ | 406.51 | 0.026 | | |
| $N_1 \rightarrow D_6$ | 406.53 | 0.048 | | |
| $N_3 \rightarrow D_2/D_{12}$ | 406.75 | 0.582 | | |
| $N_4 \rightarrow \pi_4^*$ | 406.84 | 0.304 | | |
| $N_2 \rightarrow D_3$ | 407.00 | 0.156 | | |
| $N_1 \rightarrow D_2$ | 407.13 | 0.120 | | |
| $N_1 \rightarrow D_5$ | 407.37 | 0.008 | | |

Table S14: N K-edge XAS transition energies and relative intensities (with respect to the strongest transition) of adenine, computed using IVO-GASCI-DSRG-PT3 with the cc-pVQZ basis set. The experimental frequencies[4] are also listed. The theoretical frequencies are shifted by −1.6 eV to align with the experimental results.

| Transition | Theory | | Experiment | |
|---|---|---|---|---|
| | $\omega$ (eV) | $f^{\mathrm{rel}}$ | Maximum | $\omega$ (eV) |
| $N_2 \rightarrow D_9$ | 407.51 | 0.003 | | |
| $N_1 \rightarrow D_8$ | 407.81 | 0.000 | | |
| $N_2 \rightarrow D_1$ + HOMO$\rightarrow \pi_1^*$ | 407.88 | 0.000 | | |
| $N_1 \rightarrow \pi_1^*$ + HOMO$\rightarrow \pi_1^*$ | 408.05 | 0.107 | | |
| $N_1 \rightarrow D_{10}$ | 408.20 | 0.507 | | |
| $N_2 \rightarrow D_{12}$ | 408.23 | 0.252 | | |
| $N_1 \rightarrow D_1$ + HOMO$\rightarrow \pi_1^*$ | 408.47 | 0.001 | | |
| $N_2 \rightarrow \pi_1^*$ + HOMO$\rightarrow \pi_1^*$ | 408.61 | 0.005 | | |
| $N_1 \rightarrow D_7$ | 408.71 | 0.265 | | |
| $N_2 \rightarrow D_4$ | 408.81 | 0.736 | | |
| $N_1 \rightarrow \pi_3^*$ | 408.87 | 0.160 | | |

Table S15: C K-edge XAS transition energies and relative intensities (with respect to the strongest transition) of adenine, computed using IVO-GASCI-DSRG-PT3 with the cc-pVQZ basis set. The experimental frequencies[4] are also listed. The theoretical frequencies are shifted by −1.0 eV to align with the experimental results.

| Transition | Theory | | Experiment | |
|---|---|---|---|---|
| | $\omega$ (eV) | $f^{\text{rel}}$ | Maximum | $\omega$ (eV) |
| $C_{10}$ 1s $\rightarrow$ $\pi_2^*$ | 285.81 | 0.050 | | |
| $C_{10}$ 1s $\rightarrow$ $\pi_1^*$ | 286.14 | 0.416 | A | 286.0 |
| $C_9$ 1s $\rightarrow$ $\pi_1^*$ | 286.36 | 0.926 | A′ | 286.4 |
| $C_7$ 1s $\rightarrow$ $\pi_1^*$ | 286.52 | 0.890 | B | 286.8 |
| $C_8$ 1s $\rightarrow$ $\pi_1^*$ | 287.33 | 0.919 | | |
| $C_6$ 1s $\rightarrow$ $\pi_1^*$ | 287.45 | 1.000 | C | 287.4 |
| $C_{10}$ 1s $\rightarrow$ $\pi_3^*$ | 287.62 | 0.145 | C′ | 288.0 |
| $C_8$ 1s $\rightarrow$ $\pi_2^*$ | 287.71 | 0.023 | | |
| $C_9$ 1s $\rightarrow$ $\pi_2^*$ | 287.74 | 0.050 | | |
| $C_7$ 1s $\rightarrow$ $\pi_2^*$ | 288.33 | 0.005 | | |
| $C_{10}$ 1s $\rightarrow$ $D_4$ | 288.44 | 0.007 | | |
| $C_7$ 1s $\rightarrow$ $\pi_4^*$ | 288.87 | 0.079 | | |
| $C_{10}$ 1s $\rightarrow$ $D_5$ | 288.99 | 0.023 | | |
| $C_6$ 1s $\rightarrow$ $\pi_2^*$ | 289.10 | 0.009 | | |
| $C_9$ 1s $\rightarrow$ $\pi_4^*$ | 289.15 | 0.046 | | |
| $C_8$ 1s $\rightarrow$ $\pi_3^*$ | 289.24 | 0.064 | | |
| $C_7$ 1s $\rightarrow$ $D_1$ | 289.31 | 0.214 | D | 289.0 |
| $C_{10}$ 1s $\rightarrow$ $D_5/D_3$ | 289.64 | 0.019 | | |
| $C_9$ 1s $\rightarrow$ $D_5$ | 289.92 | 0.033 | | |
| $C_{10}$ 1s $\rightarrow$ $D_7$ | 289.95 | 0.001 | | |
| $C_8$ 1s $\rightarrow$ $D_3$ | 290.16 | 0.025 | | |
| $C_{10}$ 1s $\rightarrow$ $D_6$ | 290.20 | 0.010 | | |
| $C_7$ 1s $\rightarrow$ $D_3$ | 290.30 | 0.085 | | |
| $C_9$ 1s $\rightarrow$ $D_1$ | 290.32 | 0.254 | | |
| $C_6$ 1s $\rightarrow$ $\pi_3^*$ | 290.42 | 0.122 | | |
| $C_7$ 1s $\rightarrow$ $D_5/D_6/D_7$ | 290.56 | 0.032 | | |
| $C_9$ 1s $\rightarrow$ $D_6/D_2$ | 290.59 | 0.049 | | |
| $C_9$ 1s $\rightarrow$ $\pi_1^*$ + HOMO $\rightarrow$ $\pi_1^*$ | 290.66 | 0.048 | | |
| $C_{10}$ 1s $\rightarrow$ $D_8$ | 290.70 | 0.024 | | |
| $C_8$ 1s $\rightarrow$ $D_4$ | 290.88 | 0.004 | | |
| $C_9$ 1s $\rightarrow$ $\pi_1^*$ + HOMO $\rightarrow$ $\pi_1^*$ | 290.96 | 0.002 | | |
| $C_{10}$ 1s $\rightarrow$ $\pi_4^*$ | 291.03 | 0.576 | | |

Table S15: C K-edge XAS transition energies and relative intensities (with respect to the strongest transition) of adenine, computed using IVO-GASCI-DSRG-PT3 with the cc-pVQZ basis set. The experimental frequencies[4] are also listed. The theoretical frequencies are shifted by −1.0 eV to align with the experimental results.

| Transition | Theory | | Experiment | |
|---|---|---|---|---|
| | $\omega$ (eV) | $f^{\text{rel}}$ | Maximum | $\omega$ (eV) |
| $C_9$ 1s $\to$ $D_6$ | 291.12 | 0.021 | | |
| $C_7$ 1s $\to$ $D_5/D_6/D_8$ | 291.21 | 0.001 | | |
| $C_9$ 1s $\to$ $\pi_4^*$ | 291.35 | 0.011 | | |
| $C_{10}$ 1s $\to$ $D_{10}$ | 291.38 | 0.189 | | |
| $C_6$ 1s $\to$ $D_6$ | 291.39 | 0.006 | | |
| $C_8$ 1s $\to$ $D_2$ | 291.41 | 0.025 | | |
| $C_7$ 1s $\to$ $\pi_1^*$ + HOMO $\to \pi_1^*$ | 291.43 | 0.031 | | |
| $C_6$ 1s $\to$ $\pi_1^*$ + HOMO $\to \pi_1^*$ | 291.53 | 0.196 | | |
| $C_9$ 1s $\to$ $\pi_1^*$ + HOMO $\to \pi_1^{*a}$ | 291.61 | 0.129 | | |
| $C_6$ 1s $\to$ $\pi_1^*$ + HOMO $\to \pi_1^*$ | 291.69 | 0.008 | | |
| $C_8$ 1s $\to$ $D_5$ | 291.70 | 0.034 | | |
| $C_8$ 1s $\to$ $\pi_1^*$ + HOMO $\to \pi_1^*$ | 291.70 | 0.005 | | |
| $C_{10}$ 1s $\to$ $D_{12}$ | 291.72 | 0.013 | | |
| $C_7$ 1s $\to$ $\pi_4^*$ | 291.79 | 0.001 | | |
| $C_6$ 1s $\to$ $D_1$ | 291.87 | 0.060 | | |
| $C_6$ 1s $\to$ $\pi_1^*$ + HOMO $\to \pi_1^*$ | 291.92 | 0.103 | | |
| $C_7$ 1s $\to$ $D_4$ | 291.99 | 0.022 | | |
| $C_8$ 1s $\to$ $D_8$ | 292.02 | 0.042 | | |
| $C_7$ 1s $\to$ $\pi_1^*$ + HOMO $\to \pi_1^*$ | 292.05 | 0.002 | | |
| $C_{10}$ 1s $\to$ $D_{11}$ | 292.08 | 0.240 | | |
| $C_9$ 1s $\to$ $\pi_2^*$ + HOMO $\to \pi_1^*$ | 292.09 | 0.003 | | |
| $C_9$ 1s $\to$ $D_9$ | 292.13 | 0.013 | | |
| $C_8$ 1s $\to$ $\pi_5^*$ | 292.26 | 0.103 | | |
| $C_{10}$ 1s $\to$ $\pi_4^*/\pi_5^*$ | 292.38 | 0.219 | | |
| $C_7$ 1s $\to$ $\pi_1^*$ + HOMO $\to \pi_1^*$ | 292.41 | 0.002 | | |
| $C_7$ 1s $\to$ $D_9$ | 292.50 | 0.018 | | |
| $C_6$ 1s $\to$ $D_4$ | 292.55 | 0.010 | | |
| $C_8$ 1s $\to$ $D_9/D_2$ | 292.58 | 0.073 | | |
| $C_{10}$ 1s $\to$ $\pi_1^*$ + HOMO $\to \pi_1^*$ | 292.61 | 0.002 | | |
| $C_6$ 1s $\to$ $\pi_1^*$ + HOMO $\to \pi_1^*$ | 292.67 | 0.031 | | |
| $C_8$ 1s $\to$ $\pi_1^*$ + HOMO $\to \pi_1^*$ | 292.79 | 0.000 | | |
| $C_9$ 1s $\to$ $\pi_1^*$ + HOMO $\to \pi_1^*$ | 292.81 | 0.064 | | |

Table S15: C K-edge XAS transition energies and relative intensities (with respect to the strongest transition) of adenine, computed using IVO-GASCI-DSRG-PT3 with the cc-pVQZ basis set. The experimental frequencies[4] are also listed. The theoretical frequencies are shifted by −1.0 eV to align with the experimental results.

| Transition | Theory | | Experiment | |
|---|---|---|---|---|
| | $\omega$ (eV) | $f^{\text{rel}}$ | Maximum | $\omega$ (eV) |
| $C_6$ 1s $\rightarrow$ $D_9$ | 292.83 | 0.007 | | |
| $C_6$ 1s $\rightarrow$ $D_8$ | 292.95 | 0.041 | | |
| $C_7$ 1s $\rightarrow$ $\pi_3^*$ + HOMO $\rightarrow$ $\pi_1^{*b}$ | 292.97 | 0.308 | | |
| $C_9$ 1s $\rightarrow$ $D_7$ | 293.03 | 0.018 | | |
| $C_8$ 1s $\rightarrow$ $D_{12}$ | 293.12 | 0.129 | | |
| $C_6$ 1s $\rightarrow$ $\pi_2^*$ + HOMO $\rightarrow$ $\pi_2^*$ | 293.18 | 0.008 | | |
| $C_7$ 1s $\rightarrow$ $D_4$ + HOMO $\rightarrow$ $\pi_1^*$ | 293.25 | 0.118 | | |
| $C_8$ 1s $\rightarrow$ $\pi_5^{*c}$ | 293.35 | 0.250 | | |
| $C_8$ 1s $\rightarrow$ $D_9$ | 293.39 | 0.203 | | |
| $C_6$ 1s $\rightarrow$ $\pi_2^*$ + HOMO $\rightarrow$ $\pi_2^*$ | 293.49 | 0.001 | | |
| $C_6$ 1s $\rightarrow$ $D_8/D_7$ | 293.56 | 0.006 | | |

[a] 8.1% $C_9$ $\rightarrow$ $\pi_1^*$ in the wavefunction.

[b] 10.7% $C_7$ $\rightarrow$ $\pi_1^*$ in the wavefunction.

[c] 10.1% $C_8$ $\rightarrow$ $\pi_1^*$ in the wavefunction.

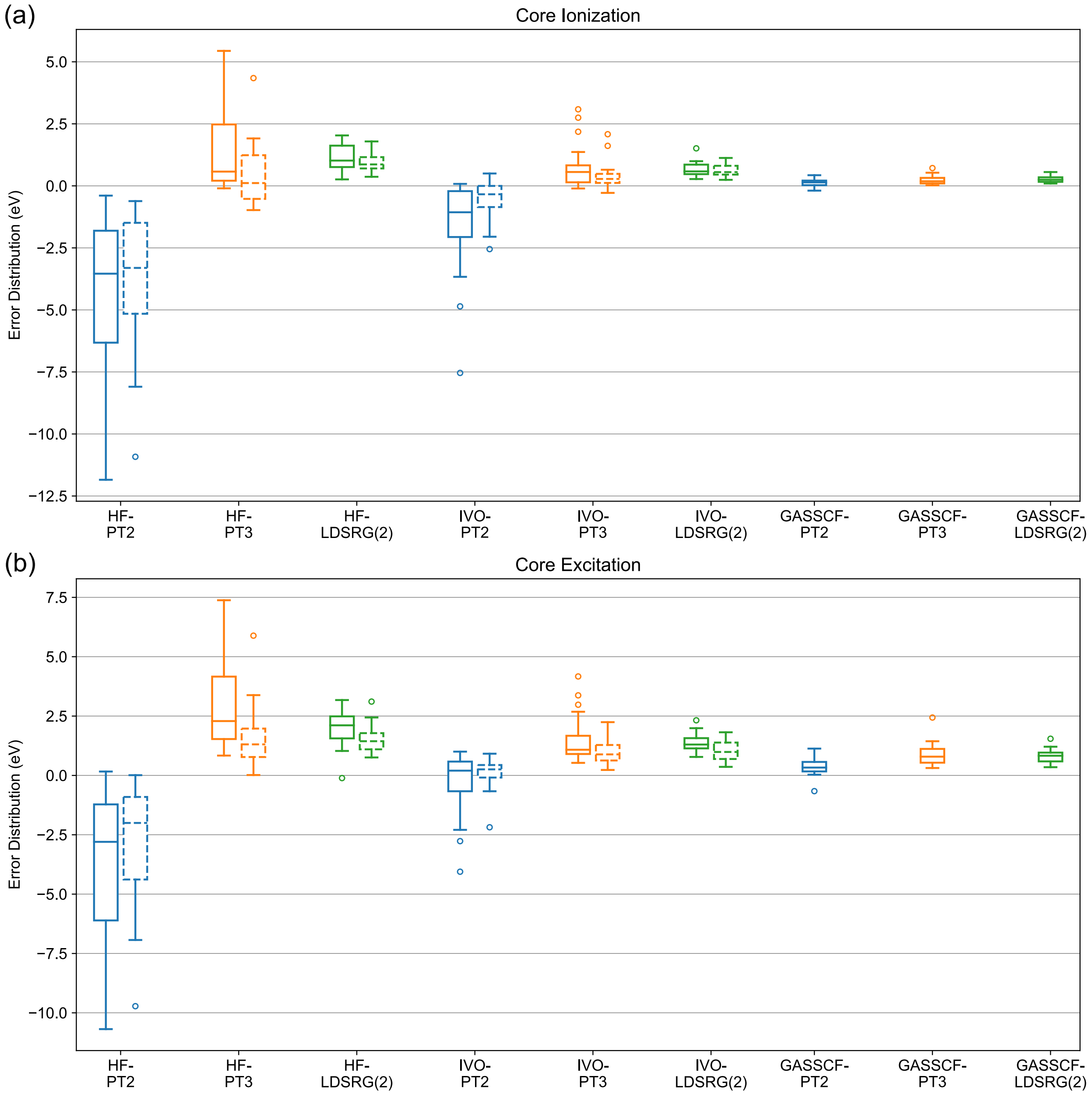


Figure S1: Error distribution (refer to experiment results) on (a) core-ionization (b) core-excitation energies of GASCI-DSRG theories based on different orbitals. Core ionization and core-excited energies were computed with a GASCI reference based on different orbitals (HF, IVO, GASSCF) and different levels of theories [PT2 = DSRG-MRPT2, PT3 = DSRG-MRPT3, LDSRG(2) = MR-LDSRG(2)] using the cc-pCVQZ-DK basis and a scalar X2C relativistic correction. Results from a small active space with 2 GAS spaces (solid) and a relative large active space with 3 GAS spaces (dash) are both reported for calculations using HF and IVO orbitals. For all GASSCF results, the orbitals are separately optimized for ground and core-ionized/core-excited states.

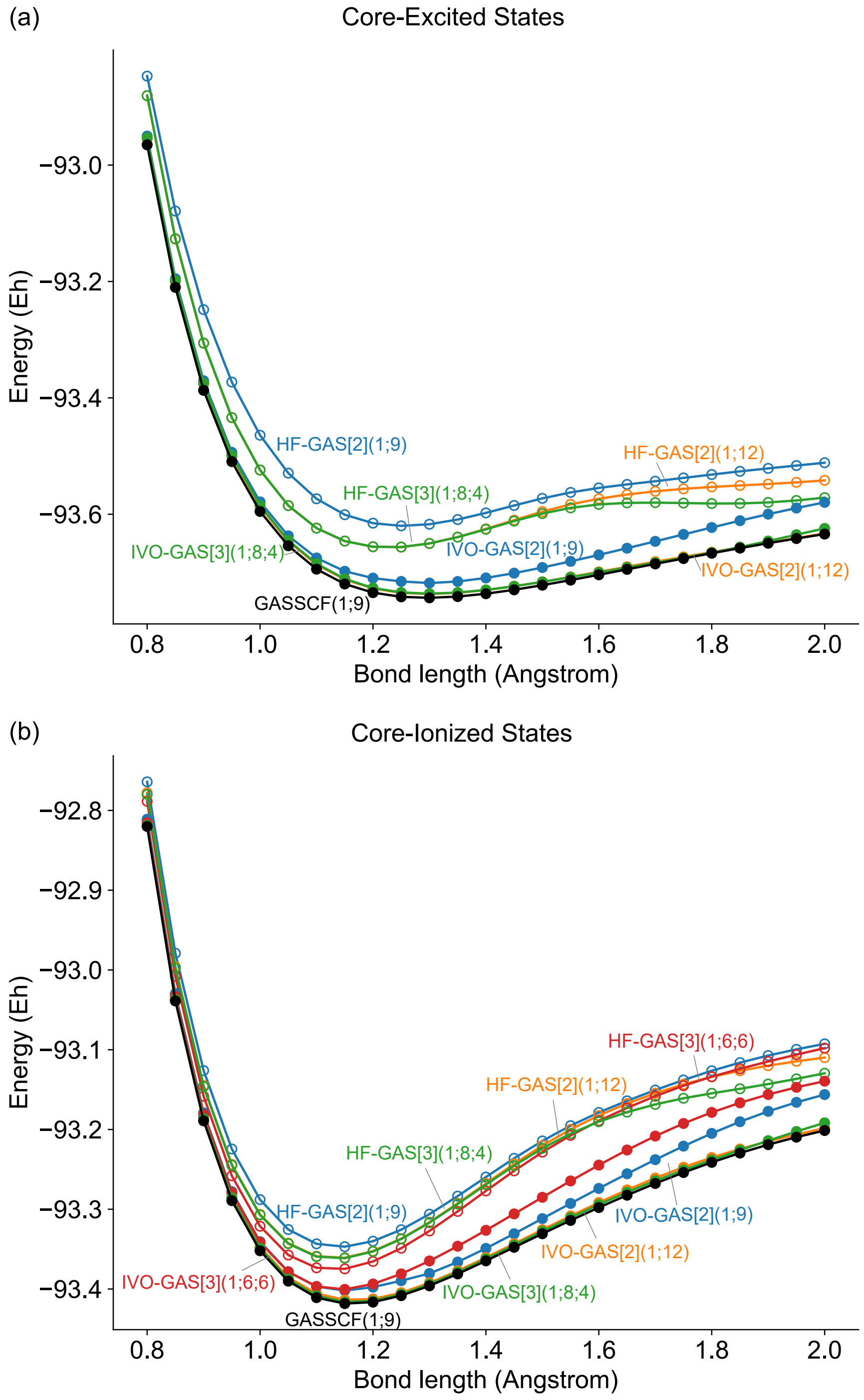


Figure S2: Potential energy surfaces of (a) the CO O 1s$\rightarrow\pi^*$ core-excited state and (b) the CO O 1s core-ionized state computed using GASCI-DSRG-MRPT3 theory based on different orbitals and choices of active spaces. The GASCI-DSRG-MRPT3 calculations use HF orbitals (open circles) and IVO orbitals (closed circles) under different GAS spaces and restrictions. Potential energy curves are calculated between 0.8 and 2.0 Å with a 0.05 Å spacing, using the cc-pCVQZ-DK basis and X2C one-electron scalar relativistic corrections.

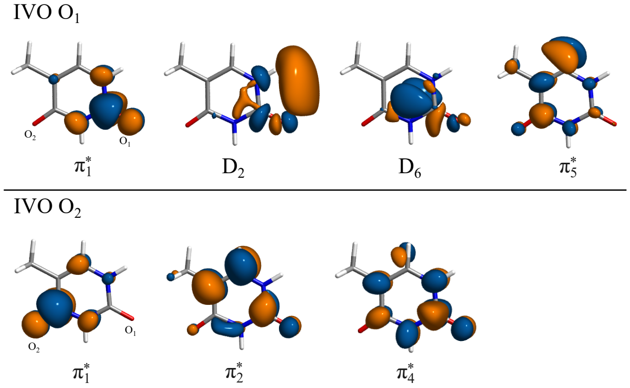


Figure S3: The valence orbitals for thymine O K-edge XAS transitions with significant relative intensities ($f^{\mathrm{rel}} > 0.10$). The calculations are performed using IVO-GASCI-DSRG-PT3 theory with the cc-pVQZ basis set.

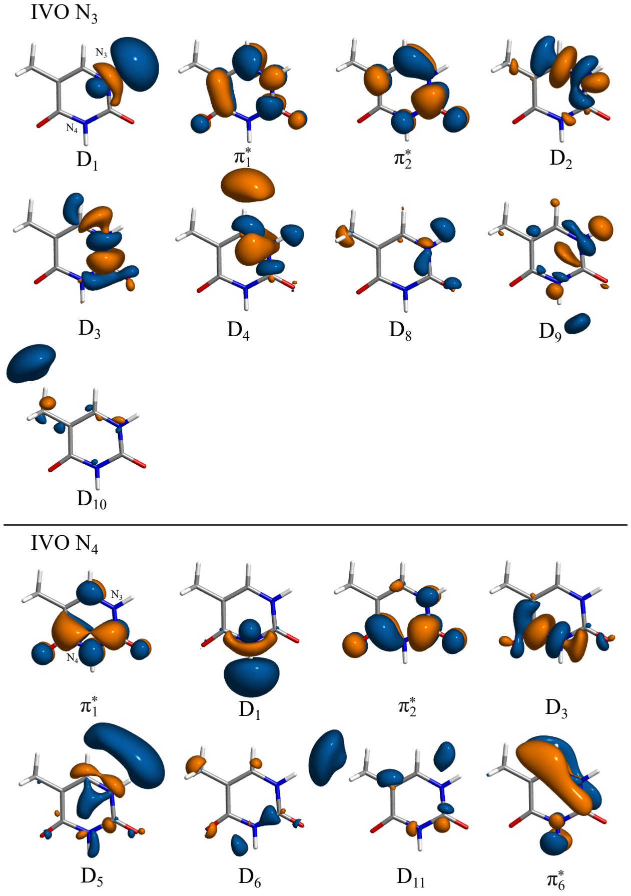


Figure S4: The valence orbitals for thymine N K-edge relative transitions with significant relative intensities ($f^{\mathrm{rel}} > 0.10$). The calculations are performed using IVO-GASCI-DSRG-PT3 theory with the cc-pVQZ basis set.

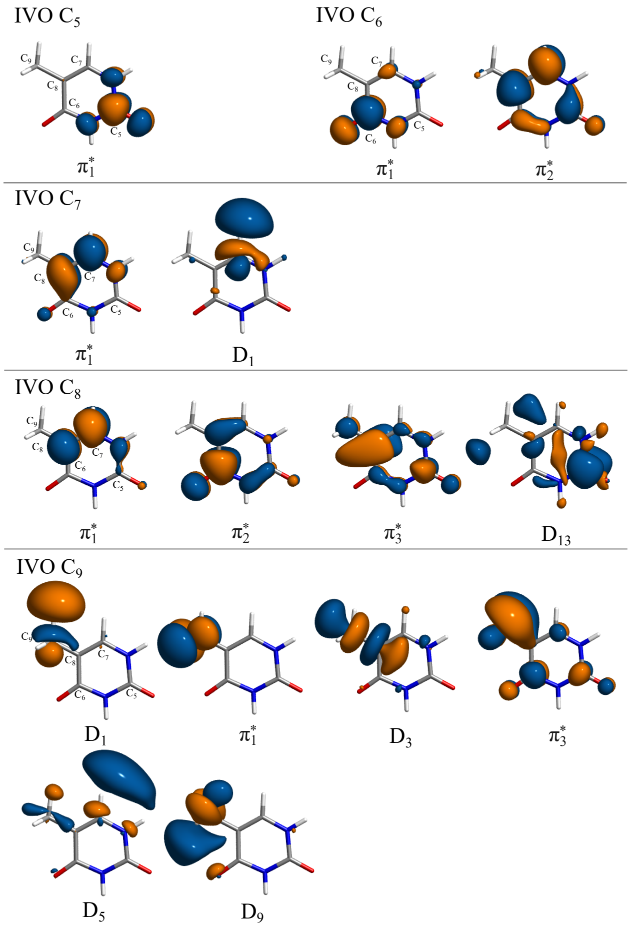


Figure S5: The valence orbitals for thymine C K-edge relative transitions with significant relative intensities ($f^{\mathrm{rel}} > 0.10$). The calculations are performed using IVO-GASCI-DSRG-PT3 theory with the cc-pVQZ basis set.

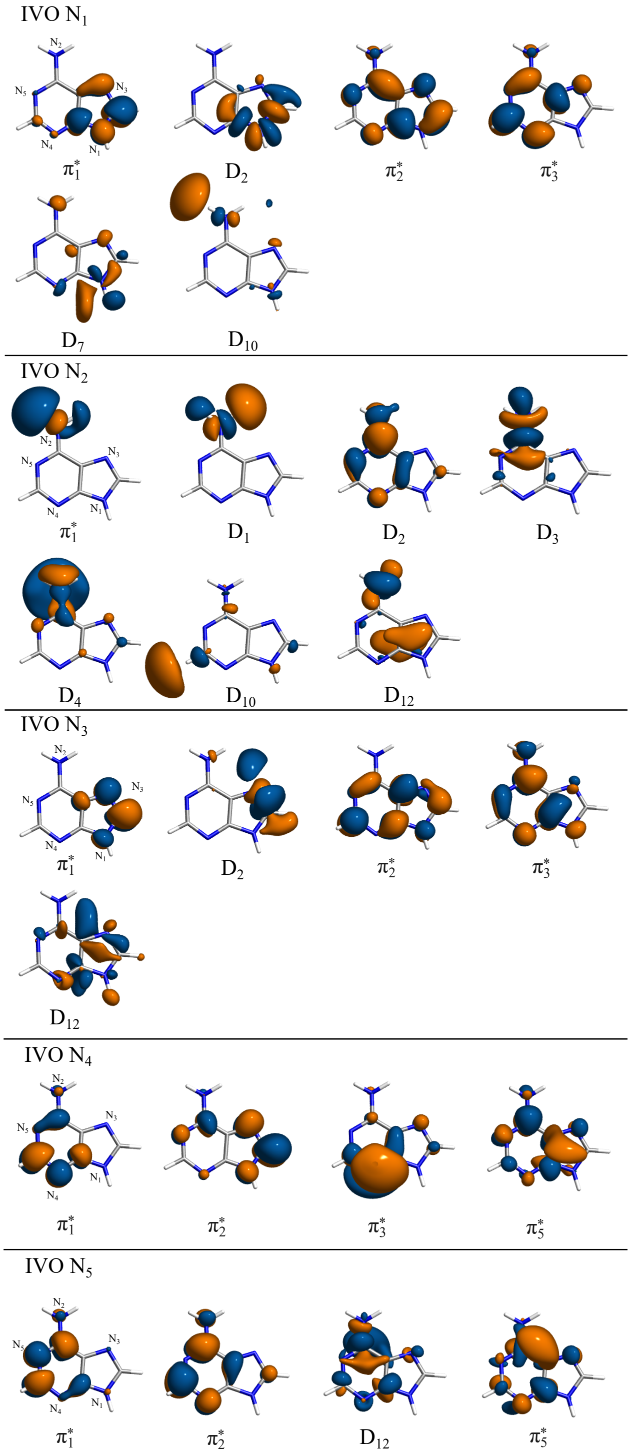


Figure S6: The valence orbitals for thymine N K-edge relative transitions with significant relative intensities ($f^{\text{rel}} > 0.10$). The calculations are performed using IVO-GASCI-DSRG-PT3 theory with the cc-pVQZ basis set.

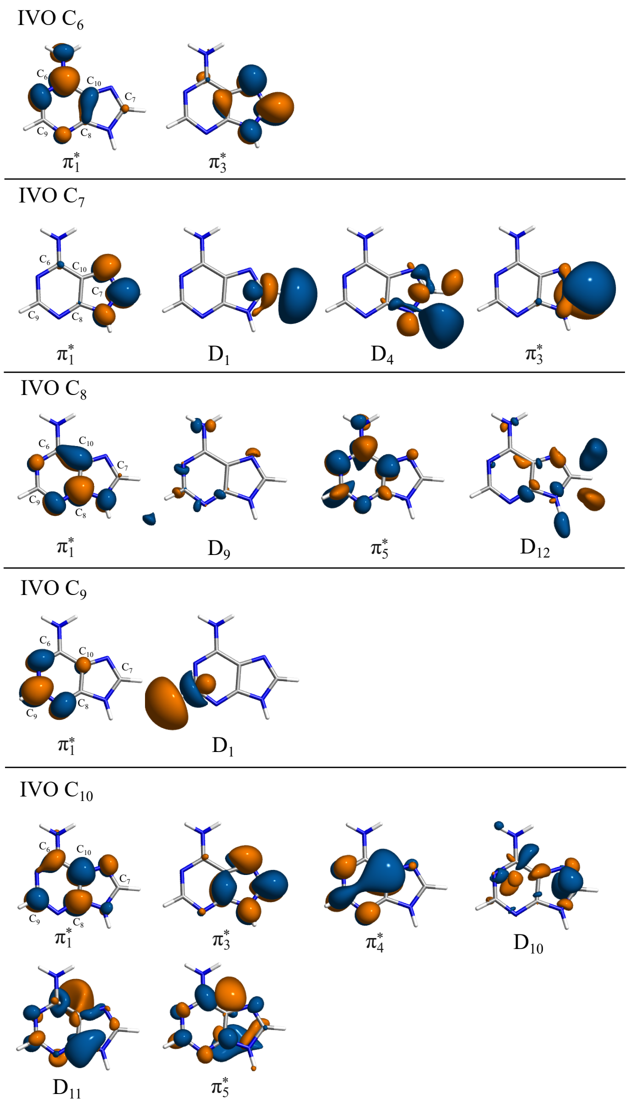


Figure S7: The valence orbitals for thymine C K-edge relative transitions with significant relative intensities ($f^{\mathrm{rel}} > 0.10$). The calculations are performed using IVO-GASCI-DSRG-PT3 theory with the cc-pVQZ basis set.

# References

(1) Huang, M.; Evangelista, F. A. A study of core-excited states of organic molecules computed with the generalized active space driven similarity renormalization group. *J. Chem. Phys.* **2023**, *158*, 124112.

(2) Golze, D.; Keller, L.; Rinke, P. Accurate Absolute and Relative Core-Level Binding Energies from GW. *J. Phys. Chem. Lett.* **2020**, *11*, 1840–1847.

(3) Fransson, T.; Brumboiu, I. E.; Vidal, M. L.; Norman, P.; Coriani, S.; Dreuw, A. XABOOM: An X-ray Absorption Benchmark of Organic Molecules Based on Carbon, Nitrogen, and Oxygen 1s $\rightarrow \pi^*$ Transitions. *J. Chem. Theory Comput.* **2021**, *17*, 1618–1637.

(4) Plekan, O.; Feyer, V.; Richter, R.; Coreno, M.; de Simone, M.; Prince, K. C.; Trofimov, A. B.; Gromov, E. V.; Zaytseva, I. L.; Schirmer, J. A theoretical and experimental study of the near edge X-ray absorption fine structure (NEXAFS) and X-ray photoelectron spectra (XPS) of nucleobases: Thymine and adenine. *Chem. Phys.* **2008**, *347*, 360–375.
